# Information measures and design issues in the study of mortality deceleration: findings for the gamma-Gompertz model

**DOI:** 10.1007/s10985-021-09518-4

**Published:** 2021-02-25

**Authors:** Marie Böhnstedt, Jutta Gampe, Hein Putter

**Affiliations:** 1 Max Planck Institute for Demographic Research, Rostock, Germany; 2 Department of Biomedical Data Sciences, Leiden University Medical Center, Leiden, The Netherlands

**Keywords:** Design, Fisher information, Gamma-Gompertz model, Likelihood ratio test

## Abstract

Mortality deceleration, or the slowing down of death rates at old ages, has been repeatedly investigated, but empirical studies of this phenomenon have produced mixed results. The scarcity of observations at the oldest ages complicates the statistical assessment of mortality deceleration, even in the parsimonious parametric framework of the gamma-Gompertz model considered here. The need for thorough verification of the ages at death can further limit the available data. As logistical constraints may only allow to validate survivors beyond a certain (high) age, samples may be restricted to a certain age range. If we can quantify the effects of the sample size and the age range on the assessment of mortality deceleration, we can make recommendations for study design. For that purpose, we propose applying the concept of the Fisher information and ideas from the theory of optimal design. We compute the Fisher information matrix in the gamma-Gompertz model, and derive information measures for comparing the performance of different study designs. We then discuss interpretations of these measures. The special case in which the frailty variance takes the value of zero and lies on the boundary of the parameter space is given particular attention. The changes in information related to varying sample sizes or age ranges are investigated for specific scenarios. The Fisher information also allows us to study the power of a likelihood ratio test to detect mortality deceleration depending on the study design. We illustrate these methods with a study of mortality among late nineteenth-century French-Canadian birth cohorts.

**Supplementary Information:**

The online version supplementary material available at 10.1007/s10985-021-09518-4.

## Introduction

Accurately describing, understanding, and, finally, projecting the trajectory of human mortality over age is crucial for assessing the future of human longevity, but it is also important in actuarial sciences, population forecasting and health care planning. The scientific modeling of human mortality over age has a long tradition. Around two centuries ago, Benjamin Gompertz published his finding that the death rates of humans increase exponentially from mid-life ages onwards (Gompertz 1825), and this regularity has since been confirmed time and time again in many populations, epochs, and circumstances. Recently, however, improved and more accurate vital registration has revealed that the increase in death rates slows down at higher ages (see, for example, Thatcher et al. 1998; Thatcher 1999). This decrease in the increase of death rates at older ages is termed *mortality deceleration*.

An explanation for this initially perplexing observation was provided early on by Beard (1959) via the so-called heterogeneity hypothesis. If the individuals in a birth cohort are subjected to non-identical mortality risks, then those with higher risks tend to die earlier, resulting in an increasingly selected group of survivors with lower mortality risks. Hence, even if the individual hazards increase exponentially, the population hazard will increase more slowly (Vaupel et al. 1979).

Although this explanation is plausible, empirical investigations have repeatedly produced mixed results (Bebbington et al. 2014). While some studies have found evidence of a downward deviation from the exponential hazard at the oldest ages (Feehan 2018), others have suggested that exponential growth continues even through advanced ages (Gavrilov and Gavrilova 2019).

The empirical study of mortality deceleration is complicated by several issues. It is a phenomenon that manifests in the tail of the life span distribution where observations necessarily become sparse, even for sizable cohorts. Whether we are able to detect mortality deceleration will depend on the actual strength of the effect and the size of the sample.

The Gompertz model originated as an actuarial device, but its ability to capture the age-trajectory of adult mortality in a multitude of circumstances prompted numerous attempts to find underlying mechanisms that would produce exponentially increasing hazards. Most attempts come from reliability theory (Gavrilov and Gavrilova 2001) and the biology of aging (see Kirkwood 2015, and references therein). Whether and which of the mechanisms will eventually apply is still an open question, however, the repeatedly confirmed exponential increase of mortality over much of the adult life span established the Gompertz model in demography, biology, and epidemiology.

When examining mortality deceleration, we have to decide over what age range death rates should be analyzed in order to uncover potential deviations from a Gompertz hazard. On the one hand, using a rather wide age range—that is, starting from relatively young ages—may run the risk that the observations at younger ages dominate the analysis, and thus mask the deceleration that is based on relatively fewer observations at older ages. This line of thought suggests that observations of higher ages at death might be more informative about a potential deceleration than observations of younger ages at death. On the other hand, using a wider age range might enable us to detect deviations from the exponential increase of the hazard early. Moreover, using a wider age range yields a larger sample size, and can increase the precision of the parameter estimates, particularly of the parameters describing the exponential increase. This might enable us to detect more easily deviations from it at higher ages. How the trade-off between these two opposing effects would play out is not clear.

Another important aspect in all studies involving old-age mortality is data quality. In particular, age misreporting is known to induce a downward bias of mortality at advanced ages (Preston et al. 1999). Therefore, scientific age validation is indispensable in studies involving individuals of very high ages (Jeune and Vaupel 1999). In practice, performing such individual checks is costly and time-consuming, and logistics can limit the number of cases that can be verified. In the application presented in Sect. 6, the ages at death could be validated for individual members of French-Canadian birth cohorts (born 1880–1896) who survived to age 90 or older. Since extending the age range by another, say, five years, to ages 85 and above, would imply a drastic increase in the number of cases to be validated, a practically relevant question is how much the extra effort would expand the information about mortality deceleration in the resulting larger dataset.

All of the considerations discussed above are questions related to optimal design. While the theory of optimal design is applied in various research fields (see Berger and Wong 2009, and the references therein), applications are less numerous in the area of survival analysis. Hwang and Brookmeyer (2003) attempted to find the optimal spacing between consecutive waves of a panel study. Becker et al. (1989) and Konstantinou et al. (2015) discussed optimal covariate settings in proportional hazards models, and McGree and Eccleston (2010) investigated the design aspects of covariates and sample size in accelerated failure-time models. Here, we will study the effects of the sample size and the age range covered by a dataset on the assessment of mortality deceleration; specifically, on the downward deviation from a Gompertz hazard.

The most commonly used approaches for describing individually heterogeneous death risks are proportional hazards frailty models (Vaupel et al. 1979; Duchateau and Janssen 2008; Balan and Putter 2020). In this paper, we focus on one specific model from this class, the gamma-Gompertz model. The individuals share an exponentially increasing Gompertz baseline hazard, but a multiplicative gamma-distributed random effect (the frailty) introduces heterogeneity of the individual mortality risks. The amount of heterogeneity is determined by the frailty variance. If the frailty variance is zero the population hazard will follow the exponential Gompertz trajectory, while a positive frailty variance implies that the population hazard decelerates at older ages. Consequently, the statistical assessment of mortality deceleration in the gamma-Gompertz model is reduced to inference about the frailty variance. In particular, the likelihood ratio test for a zero frailty variance is a commonly used approach to assess this phenomenon. However, zero is a boundary point of the parameter space for the variance parameter which violates the usual regularity assumptions. Consequently, standard asymptotic results are not directly applicable.

In this paper, we propose using the concepts of the Fisher information and of optimal design to address issues that arise in planning and evaluating studies that assess mortality deceleration in the setting of the gamma-Gompertz model. Within the likelihood framework, the Fisher information measures the amount of information about the model parameters that is contained in the data (Lehmann 1999). Therefore, the Fisher information can serve as a basis for identifying optimal designs that maximize the information about the model parameters.

The paper is organized as follows. Section 2 lays out the framework for our study by formally introducing the gamma-Gompertz model, as well as the general concepts of the Fisher information and of optimal designs. In Sect. 3, we present the Fisher information and a specific information measure for the gamma-Gompertz model, and relate them to the power of the likelihood ratio test to detect mortality deceleration. In Sect. 4, we discuss in detail the design issues that arise in studies of mortality deceleration. In Sect. 5, we assess the effects of different design choices on the information measure, and on the power of the test for specific scenarios. In Sect. 6, we apply the proposed concepts and methods to a French-Canadian mortality dataset. In Sect. 7, we conclude with a discussion of our findings.

## Framework: gamma-Gompertz model, Fisher information and study design

### Gamma-Gompertz model

We consider a continuous random variable *X* that describes adult lifespans (above some young adult age, such as 30). Its distribution is determined by the hazard function

$$\begin{aligned} h(x)=\lim _{\Delta x \searrow 0} \frac{\mathrm {P}(x<X\le x+\Delta x \mid X>x)}{\Delta x}. \end{aligned}$$

The heterogeneity hypothesis can be formalized in frailty proportional hazards models of the form $h(x\mid Z=z) = z\cdot h_0(x)$. The unobserved heterogeneity of the individuals is modeled via the positive random effect *Z* that affects a common baseline hazard $h_0(x)$ in a multiplicative way. Individuals with higher values *z* have a higher risk at any age *x*, as specified by the conditional hazard $h(x\mid Z=z)$; thus, *Z* is called the frailty.

A popular choice for the distribution of frailties is the gamma distribution. It leads to closed-form expressions for marginal survival and hazard functions. Furthermore, for the gamma distribution the frailty among survivors at any age $x >0$ again is gamma distributed, only with different parameters (Vaupel et al. 1979; Hougaard 1984; Economou and Caroni 2008). Moreover, Abbring and van den Berg (2007) showed that even if the frailty at $x=0$ is not gamma distributed the frailty among survivors converges with increasing *x* to a gamma distribution for many proportional hazards frailty models.

As the name suggests, in the gamma-Gompertz model the baseline hazard has an exponentially increasing Gompertz form, $h_0(x)=a e^{bx}$. Here the parameter $a>0$ represents the initial level of mortality for $x=0$ and $b>0$ is the rate of ageing. The frailty is gamma distributed, with a mean of one and a variance of $\sigma ^2$. The heterogeneity in frailty, and, hence, in mortality risks, is measured by the variance parameter $\sigma ^2$. In a heterogeneous population with $\sigma ^2>0$, there is a tendency of individuals with higher frailty values to die at younger ages, such that the population of survivors to higher ages consists mainly of individuals with lower mortality risks. Therefore, the marginal hazard,

1 $$\begin{aligned} h(x)=\frac{a e^{bx}}{1+\sigma ^2 \frac{a}{b}(e^{bx}-1)}, \end{aligned}$$

shows a downward deviation from the exponential increase at higher ages. In a homogeneous population with $\sigma ^2=0$, there is no such selection effect, and the marginal hazard is again of the Gompertz form, $h(x)=a e^{bx}$. Thus, the presence or the absence of mortality deceleration is determined by the parameter $\sigma ^2$ and can, for instance, be assessed by a likelihood ratio test for $H_0\!: \sigma ^2=0$ against $H_1\!: \sigma ^2>0$.

While the parameter $\sigma ^2$ describes the heterogeneity in frailty and mortality risks at the starting age of the model—that is, at $x=0$—the increasingly selected population of survivors to higher ages will be less heterogeneous in terms of their frailty and mortality risks. For example, the heterogeneity in mortality risks will be lower in the subset of survivors to ages 90 and above than among the survivors to ages 80 and above. Consequently, the age range covered by a dataset will affect the ability to assess the frailty variance, and, hence, mortality deceleration.

It is important to note that the frailty variance $\sigma ^2$ takes a value on the boundary of its parameter space if there is no heterogeneity ($\sigma ^2=0$). As this violates common regularity assumptions, some standard asymptotic results for likelihood inference might not hold, which will also affect the interpretation of the information measures in the following.

### The Fisher information

We briefly recap the concept of the Fisher information, and refer to Chapter 7 in Lehmann (1999) for further details. For a random variable *X* with density $f_X(\cdot ;\varvec{\theta })$ and parameter vector $\varvec{\theta }=(\theta _1,\theta _2,\ldots ,\theta _K)^T$, the Fisher information matrix ${\varvec{I}}(\varvec{\theta })$ is defined as

2 $$\begin{aligned} {\varvec{I}}(\varvec{\theta })={\mathbb {E}}\left[ \left( \frac{\partial }{\partial \varvec{\theta }} \ln f_X(X;\varvec{\theta })\right) \left( \frac{\partial }{\partial \varvec{\theta }} \ln f_X(X;\varvec{\theta })\right) ^T\right] , \end{aligned}$$

where the expectation ${\mathbb {E}}$ is with respect to the distribution of *X*. Under mild regularity conditions, expression (2) can be rewritten in terms of the second-order partial derivatives of the log-density of *X*,

3 $$\begin{aligned} {\varvec{I}}(\varvec{\theta })=-{\mathbb {E}}\left[ \frac{\partial ^2}{\partial \varvec{\theta }\partial \varvec{\theta }^T} \ln f_X(X;\varvec{\theta })\right] . \end{aligned}$$

The Fisher information ${\varvec{I}}(\varvec{\theta })$ is often interpreted as the amount of information a single observation of *X* contains about the model parameters $\varvec{\theta }$. The information $\varvec{I_n}(\varvec{\theta })$ of an iid sample $X_1,X_2,\ldots ,X_n$ of size *n* from the distribution of *X* is then *n*-times as large, $\varvec{I_n}(\varvec{\theta })=n{\varvec{I}}(\varvec{\theta })$. In many cases, the Fisher information (3) cannot be computed directly because it depends on the true unknown parameter value, or because the expectation is not analytically tractable. Thus, we often use the observed Fisher information matrix, which for an iid sample of size *n* is given by the negative second-order partial derivatives of the log-likelihood, evaluated at the maximum likelihood estimate (MLE) $\varvec{{\hat{\theta }}_n}$,

4 $$\begin{aligned} \varvec{{\mathcal {J}}}(\varvec{{\hat{\theta }}_n})=-\frac{\partial ^2}{\partial \varvec{\theta } \partial \varvec{\theta }^T}\sum _{i=1}^n \ln {f_X(X_i;\varvec{\theta })}\Big |_{\varvec{\theta }=\varvec{{\hat{\theta }}_n}}. \end{aligned}$$

The interpretation of ${\varvec{I}}(\varvec{\theta })$ as a measure of information is based on two different arguments. Analytically, the partial derivatives $\frac{\partial }{\partial \varvec{\theta }} \ln f_X(x;\varvec{\theta })=\frac{\frac{\partial }{\partial \varvec{\theta }}f_X(x;\varvec{\theta })}{f_X(x;\varvec{\theta })}$ in (2) describe the relative change of the density $f_X(\cdot ;\varvec{\theta })$ with respect to $\varvec{\theta }$ at the point *x*. If this change is large for one $\varvec{\theta _0}$, this parameter value can be better identified from a range of possible values $\varvec{\theta }$. Similarly, the second-order partial derivatives $\frac{\partial ^2}{\partial \varvec{\theta }\partial \varvec{\theta }^T} \ln f_X(X;\varvec{\theta })$ in (3) describe the curvature of the log-density $\ln f_X(\cdot ;\varvec{\theta })$ with respect to $\varvec{\theta }$, and, thus, the curvature of the contributions to the log-likelihood function. A sample for which the log-likelihood shows a clearer peak at some $\varvec{\theta _0}$, and for which this value is, therefore, more clearly distinguished from other values $\varvec{\theta }$, is viewed as more informative about the parameter than samples with a flatter log-likelihood.

A second justification for the notion of information rests on the following result for asymptotically normal estimators. If an estimator $\delta _n$ of $\theta _k$ satisfies $\sqrt{n}(\delta _n-\theta _k){\mathop {\longrightarrow }\limits ^{d}}{\mathcal {N}}(0,v(\varvec{\theta }))$, then its variance is bounded below by $[{\varvec{I}}(\varvec{\theta })]^{-1}_{kk}$, which denotes the *k*th diagonal element of the inverse of the information matrix ${\varvec{I}}(\varvec{\theta })$ (see Lehmann and Casella 1998, p. 462). In particular, the MLE $\varvec{{\hat{\theta }}_n}$ attains this lower bound under suitable regularity conditions (cf. Lehmann and Casella 1998, p. 463),

5 $$\begin{aligned} \sqrt{n}(\varvec{{\hat{\theta }}_n}-\varvec{\theta }){\mathop {\longrightarrow }\limits ^{d}}{\mathcal {N}}({\varvec{0}},[{\varvec{I}}(\varvec{\theta })]^{-1}), \end{aligned}$$

such that each ${\hat{\theta }}_{nk}$ is asymptotically efficient, $\sqrt{n}({\hat{\theta }}_{nk}-\theta _k){\mathop {\longrightarrow }\limits ^{d}}{\mathcal {N}}(0,[{\varvec{I}}(\varvec{\theta })]^{-1}_{kk})$. In this sense, a sample is more informative if the parameters can be estimated with higher precision.

In summary, following the exposition above, the Fisher information serves as a suitable measure of the information contained in a sample about the unknown parameter in likelihood-based inference.

### Optimal design

The Fisher information can be instrumental for determining optimal designs. The aim is to find a design that maximizes some scalar function of the information matrix, and that therefore maximizes the information, in a suitably defined way, about all or some particular model parameters.

Different criteria for defining and assessing the optimality of a design have been suggested (see Silvey 1980, for an early monograph, and Atkinson 1988, for an early review). If all elements of the parameter vector $\varvec{\theta }$ are of interest, two popular scalar measures of information are *D*- and *A*-optimality. A design is called *D*-optimal if the design maximizes the determinant of the information matrix, $\det ({\varvec{I}}(\varvec{\theta }))$. Alternatively, the criterion of *A*-optimality refers to the trace of the inverse information matrix $[{\varvec{I}}(\varvec{\theta })]^{-1}$ and states that a design is optimal if the design attains the maximum possible value for the inverse of this trace, that is, for $1/\mathrm {tr}([{\varvec{I}}(\varvec{\theta })]^{-1})$.

Both of these information measures are functions of the eigenvalues of the information matrix. The determinant equals the product and the trace equals the sum of the eigenvalues of a matrix, respectively; and the eigenvalues of $[{\varvec{I}}(\varvec{\theta })]^{-1}$ are the reciprocals of the eigenvalues of ${\varvec{I}}(\varvec{\theta })$. These eigenvalues of the information matrix are related to estimator precision. For an estimator $\varvec{{\hat{\theta }}_n}$ that is asymptotically normal with the covariance matrix given by the inverse Fisher information matrix $[{\varvec{I}}(\varvec{\theta })]^{-1}$ as in (5), a confidence region for the parameter vector $\varvec{\theta }$ takes the form of an ellipsoid. The axes of the ellipsoid are characterized by the eigenvalues and the eigenvectors of the matrix $[{\varvec{I}}(\varvec{\theta })]^{-1}$. More precisely, the eigenvectors determine the direction of the axes of the ellipsoid, and the eigenvalues are proportional to the squared lengths of the axes. Therefore, the size of the confidence ellipsoid and the precision of the estimator largely depend on the eigenvalues of $[{\varvec{I}}(\varvec{\theta })]^{-1}$. In particular, the volume of the confidence ellipsoid is proportional to the product of the eigenvalues of $[{\varvec{I}}(\varvec{\theta })]^{-1}$. As a consequence, maximizing the information in terms of *D*-optimality corresponds to minimizing the volume of the confidence ellipsoid.

The criteria of *D*- and *A*-optimality weigh all dimensions of the problem equally. In contrast, the criterion of *E*-optimality seeks to maximize only the smallest eigenvalue of the information matrix. This is equivalent to minimizing the largest eigenvalue of $[{\varvec{I}}(\varvec{\theta })]^{-1}$, which measures the uncertainty about the parameters in the direction of the largest axis of the confidence ellipsoid. Because the parameters are estimated with least precision in this direction, an *E*-optimal design maximizes the precision in the estimation of the least well-estimated parameter combinations.

If one particular linear combination of the parameters is of specific interest, the criterion of $D_A$-optimality is applied. If ${\varvec{A}}\varvec{\theta }$ denotes the linear combination of the parameters, where ${\varvec{A}}$ is a $p\times K$ matrix of rank $p<K$, then, by analogy with *D*-optimality, one maximizes the determinant of the inverse of ${\varvec{A}}[{\varvec{I}}(\varvec{\theta })]^{-1}{\varvec{A}}^T$. The criterion of $D_A$-optimality also allows us to focus on only one parameter $\theta _k$. For that purpose, a matrix ${\varvec{A}}$ of dimension $1\times K$ is defined with entry 1 for the *k*th element, and with zeros otherwise. The information measure then simplifies to $({\varvec{A}}[{\varvec{I}}(\varvec{\theta })]^{-1}{\varvec{A}}^T)^{-1}=1/[{\varvec{I}}(\varvec{\theta })]^{-1}_{kk}$. In a regular setting with an asymptotically normal estimator ${\hat{\theta }}_k$ that satisfies (5), maximizing the information measure $1/[{\varvec{I}}(\varvec{\theta })]^{-1}_{kk}$ is equivalent to minimizing the asymptotic variance of ${\hat{\theta }}_k$, or, in other words, maximizing its precision.

If the information matrix ${\varvec{I}}(\varvec{\theta })$ and the derived information measures depend on the unknown parameter vector $\varvec{\theta }$, these designs are said to be only locally optimal designs for the given values of the parameter(s). However, we can still evaluate the information measures over a range of possible parameter values to assess the robustness of the optimality of the design against changes in the parameter values.

## Information measures in the gamma-Gompertz model

In this section, we develop the concepts of Sects. 2.2 and 2.3 specifically for the gamma-Gompertz model. After providing details on the computation of the Fisher information matrix, we specify an information measure for $D_A$-optimality, and discuss its interpretation. In Sect. 3.4, we show that this measure also plays a role in the calculation of the power of the likelihood ratio test to detect mortality deceleration.

### Fisher information in the gamma-Gompertz model

The aim is to derive the Fisher information matrix according to (3) specifically for an observation from the gamma-Gompertz model (1). For this model, the parameter vector consists of three components: the Gompertz baseline parameters *a* and *b* and the frailty variance $\sigma ^2$, so that $\varvec{\theta }=(a,b,\sigma ^2)^T$. The density of lifespan *X* is given by

$$\begin{aligned} f_X(x;a,b,\sigma ^2)=a\,e^{bx}\,\left[ 1+\sigma ^2\frac{a}{b}(e^{bx}-1)\right] ^{-\left( 1+\frac{1}{\sigma ^2}\right) }. \end{aligned}$$

(The value $x=0$ here marks the age from which the exponentially increasing Gompertz hazard has been established as a good model for human mortality, commonly a mid-adult age such as 30 or 40.)

As is common in the analysis of time-to-event data, the observations are often subject to censoring or truncation. Left truncation occurs in our context if the data are limited to individuals who have survived beyond a certain age $\breve{x}$, as discussed in Sect. 1. In our case, this left-truncation age is identical for all individuals ($\breve{x} = 90$). Censoring occurs if some individuals are still alive at the end of follow-up. In our study, we only analyze birth cohorts who are already extinct—that is, all members have already died—and we will not consider right censoring. (For the calculation of the Fisher information with censoring and truncation for a class of location-scale distributions, see Escobar and Meeker 1998.)

For left-truncated observations, the Fisher information needs to be calculated for the truncated $(X\mid X>\breve{x})$ with density $f_{X|X>\breve{x}}(\cdot ;\varvec{\theta })=f_X(\cdot ;\varvec{\theta })/S_X(\breve{x};\varvec{\theta })$ on $(\breve{x},\infty )$, where $S_X(x;\varvec{\theta })=\mathrm {P}(X>x;\varvec{\theta })$ denotes the survival function of *X*. Consequently, formula (3) for the information matrix is adapted as

3′ $$\begin{aligned} {\varvec{I}}(\varvec{\theta })&=-{\mathbb {E}}\left[ \frac{\partial ^2}{\partial \varvec{\theta }\partial \varvec{\theta }^T}\ln {f_{X|X>\breve{x}}(X;\varvec{\theta })}\mid X>\breve{x}\right] \nonumber \\&=-\int \left( \frac{\partial ^2}{\partial \varvec{\theta }\partial \varvec{\theta }^T}\ln {f_{X|X>\breve{x}}(u;\varvec{\theta })}\right) f_{X|X>\breve{x}}(u;\varvec{\theta })\,\mathrm {d}u. \end{aligned}$$

Computing the Fisher information matrix in the gamma-Gompertz model requires the second-order partial derivatives of the log-density of the gamma-Gompertz model *X* for complete data, or of $(X\mid X>\breve{x})$ for left-truncated data with respect to the parameters. The formulas are given in Section S.1 of the supplementary material. Based on these, we obtain explicit formulas for the observed Fisher information matrix $\varvec{{\mathcal {J}}}(\varvec{{\hat{\theta }}_n})$, defined in (4), for a given sample with corresponding MLE $\varvec{{\hat{\theta }}_n}$.

In contrast, the exact calculation of the Fisher information matrix ${\varvec{I}}(\varvec{\theta })$ in (3′) requires taking the (negative) expectations of the second-order partial derivatives. As closed-form expressions for these integrals do not exist, we propose approximating the expectations using numerical integration.

In the absence of an analytical expression for the Fisher information matrix ${\varvec{I}}(\varvec{\theta })$ in the gamma-Gompertz model, there is no closed-form function of ${\varvec{I}}(\varvec{\theta })$ of the parameters $\varvec{\theta }$, the sample size *n*, and the age at left truncation $\breve{x}$. However, ${\varvec{I}}(\varvec{\theta })$ can be evaluated over a range of relevant values for these quantities in order to get an impression of how they affect the information matrix. Further computational details are given in Section S.2 of the supplementary material.

### $D_A$-optimality in the gamma-Gompertz model

In the gamma-Gompertz model, the presence or the absence of mortality deceleration is determined by the frailty variance, which also to a large extent controls how strongly the hazard decelerates. Thus, for the assessment of mortality deceleration, our main interest lies in the parameter $\sigma ^2$, while the Gompertz parameters *a* and *b* are treated as nuisance. Hence, we will evaluate designs primarily according to the criterion of $D_A$-optimality, and define the matrix ${\varvec{A}}$ from Sect. 2.3 as ${\varvec{A}}=(0,0,1)$. The resulting information measure is then $1/[{\varvec{I}}(\varvec{\theta })]^{-1}_{33}$; in the following, we will denote $[{\varvec{I}}(\varvec{\theta })]^{-1}_{33}$ as $\kappa ^2$. It is important to note that $\kappa ^2$ still depends on the true parameter value $\varvec{\theta }$, but also on the observation scheme (such as left-truncation age $\breve{x}$), although this is suppressed in the notation. A design will be preferred over another if it has a smaller $\kappa ^2$.

### Interpretation of information measures in a non-standard setting

As we noted in Sect. 2.2, the use of the Fisher information for study design can be motivated by the result that the asymptotic covariance matrix of the MLEs is given by the inverse Fisher information; see (5). This result holds under standard conditions, which are, however, violated in the present framework of the gamma-Gompertz model, because the frailty variance takes a value on the boundary of the parameter space if there is no mortality deceleration ($\sigma ^2=0$). The asymptotic distribution of the MLE in the gamma-Gompertz model was derived in Böhnstedt and Gampe (2019). For sufficiently large $\sigma ^2>0$, the MLE $\varvec{ {{\hat{\theta }}}}=({\hat{a}},{\hat{b}},{\hat{\sigma }}^2)^T$ is still asymptotically normal with covariance matrix $[\varvec{I_n}(\varvec{\theta })]^{-1}$ as in (5); but for $\sigma ^2=0$, the MLE has a two-component mixture distribution. As a result, minimizing the element $\kappa ^2$ of the inverse Fisher information in order to find an optimal design corresponds to minimizing the asymptotic variance of the parameter estimate ${\hat{\sigma }}^2$ only if the true $\sigma ^2>0$ is sufficiently large. If $\sigma ^2=0$, the quantity $n^{-1}\kappa ^2$ does not correspond to the variance of ${\hat{\sigma }}^2$.

Nonetheless, the element $\kappa ^2$ can be used for the evaluation of certain design choices—for example, for comparing different alternatives for the age range covered by a sample. In a simulation study (for details about the scenarios see Sect. 5), we found that the relative changes in $n^{-1}\kappa ^2$ were very close to the relative changes in the variance of ${\hat{\sigma }}^2$ even if $\sigma ^2=0$ (see bottom panels of Figure S.1 in the supplementary material). This finding suggests that comparative statements about the amount of information or the variance of ${\hat{\sigma }}^2$ for subsets of a sample that cover different age ranges can still be based on ratios of the corresponding $\kappa ^2$. Unfortunately, this does not apply for comparisons across different scenarios defined by different $\varvec{\theta }$.

Consequently, the quantity $\kappa ^{-2}$ should only be related to estimator variance in cases in which this is known to be appropriate. Otherwise, we should stick to the notion of a measure of information; e.g., in the sense of local curvature of the log-likelihood.

### Power of the likelihood ratio test

A common approach for assessing mortality deceleration in the framework of the gamma-Gompertz model is a likelihood ratio test for $H_0\!: \sigma ^2=0$ against $H_1\!: \sigma ^2>0$. Under the null hypothesis, the value of the variance parameter lies on the boundary of the parameter space so that the likelihood ratio test statistic is not asymptotically chi-squared distributed with one degree of freedom. Instead, one can adopt the results of Self and Liang (1987) to show that, if $H_0$ holds, the test statistic asymptotically follows a 50:50 mixture of a chi-squared distribution with one degree of freedom and a point mass at zero. Incorrectly assuming a chi-squared distribution with one degree of freedom for the test statistic implies a larger critical value, hence fewer rejections of $H_0$, and ultimately lower power to detect a positive $\sigma ^2>0$.

An explicit formula for the asymptotic power of the likelihood ratio test based on a sample from a gamma-Gompertz model with frailty variance $\sigma ^2$ was derived by Böhnstedt and Gampe (2019). According to their Lemma 6, the power $\beta _n$ of the likelihood ratio test at level $\alpha$ and sample size *n* can be approximated by

6 $$\begin{aligned} \beta _n(\sigma ^2)\approx 1-\Phi \left( \Phi ^{-1}(1-\alpha )-\frac{\sqrt{n}\sigma ^2}{\kappa }\right) , \end{aligned}$$

where $\Phi (\cdot )$ is the standard normal distribution function and $\kappa$ is the square root of the element of the inverse Fisher information, as defined above. (The proof can be found in the online supplementary material of Böhnstedt and Gampe 2019.) Thus, through $\kappa$, the power of the likelihood ratio test depends on the true parameter $\varvec{\theta }$, but also on possible left truncation; that is, on the age range of the data. Based on our computation of the Fisher information matrix and the resulting $\kappa ^2$, we can now also determine the power of the likelihood ratio test theoretically, without performing extensive simulation studies. Moreover, with regard to study design, we see from formula (6) that designs that minimize $\kappa ^2$ simultaneously maximize the power of the likelihood ratio test to detect mortality deceleration.

## Design considerations in assessing mortality deceleration

The precision of parameter estimates and the power of statistical tests to detect mortality deceleration depend on the characteristics of the dataset under study. We want to quantify the effects that the size of the sample as well as the age range that it covers have on the information contained in the data about the phenomenon. For that purpose, we denote by ${\mathcal {I}}$ a scalar measure of information that is derived from the Fisher information matrix ${\varvec{I}}(\varvec{\theta })$, such as ${\mathcal {I}}=\det ({\varvec{I}}(\varvec{\theta }))$ or ${\mathcal {I}}=\kappa ^{-2}$.

In the first part of this section, we will discuss how we can assess the effect of the age range that is covered by the data. The age range of a dataset is usually restricted because accurate age validation is required, but it is often not feasible to perform the validation for an extensive part of a birth cohort. As mortality deceleration occurs at the tail of the survival distribution, studies that examine this phenomenon focus on the older ages, and, therefore, usually collect information only on survivors beyond a certain age *x*. On the one hand, the observation of a death at older ages might be expected to carry more information about mortality deceleration than a death at younger ages. On the other hand, the continuing selection of more robust individuals with lower frailty values leads to a decrease in the variance of frailty among survivors to higher ages (Vaupel et al. 1979; Hougaard 1984; Economou and Caroni 2008). Therefore it could become more difficult to assess mortality deceleration for higher left-truncation ages. Moreover, observations of deaths at younger ages can provide indirect information about the parameter $\sigma ^2$, because they lead to increased precision in the estimation of the Gompertz parameters *a* and *b*.

To see how these effects trade off, we look at ${\mathcal {I}}_{x+}$, the information measure for an observation left-truncated at age *x* for a given $\varvec{\theta }$. The pattern of the absolute measure ${\mathcal {I}}_{x+}$ across different *x* tells us which age range is most informative. In addition, ratios like ${\mathcal {I}}_{80+}/{\mathcal {I}}_{90+}$ quantify the change in information if observations are left-truncated at an earlier age; here, at $x=80$, rather than at a later age, like $x=90$.

The Fisher information matrix ${\varvec{I}}(\varvec{\theta })$ and derived measures such as ${\mathcal {I}}=\kappa ^{-2}$ correspond to a single observation. If we want to compare the amount of information that is available in a situation in which all survivors to ages $x=80$ and above (80+) can be studied to a situation in which only survivors to ages 90+ can be studied, we should also take into account that the 80+ dataset will include more individuals than the 90+ dataset, because for studies on mortality deceleration, all members of a cohort who survive beyond a certain age will usually be included in the sample. For that purpose, we scale the information measure ${\mathcal {I}}_{x+}$ by the probability of obtaining an observation of a death at some age $x+$, and define the scaled measure as ${\mathcal {I}}^{(s)}_{x+}={\mathcal {I}}_{x+}\cdot \mathrm {P}(X>x)$.

For scenarios with sufficiently large $\sigma ^2$, the variance of ${\hat{\sigma }}^2$ can be approximated by $n^{-1}\kappa ^2$. Thus, we can also draw conclusions about the precision of the estimate of the frailty variance based on ${\mathcal {I}}=\kappa ^{-2}$. In this case, the inverse of the ratio ${\mathcal {I}}^{(s)}_{80+}/{\mathcal {I}}^{(s)}_{90+}$ describes the relative change in the variance of ${\hat{\sigma }}^2$ when data on all deaths between ages 80 and 89 could be added to a dataset that currently contains information only on all survivors to ages 90+. On the basis of such numbers, practitioners could decide whether it is worthwhile to extend an existing dataset to also include information on deaths at earlier ages.

Second, let us turn to some sample size considerations. Mortality studies are generally based on populations or a specific subset thereof, such as all survivors of a birth cohort beyond a certain age. Thus, the sample size is not actively chosen, but simply results from the size of the population under study. Nonetheless, sample size calculations are useful either for judging a priori whether a dataset provides enough information to produce meaningful results, or for adequately interpreting the results in comparative studies across different countries. Let us assume that some countries or regions are expected to have similar mortality patterns, and that mortality deceleration has been detected in one of them from an 80+ sample. The information contained in that sample is known to be $n_{80+}{\mathcal {I}}_{80+}$. Then, if for a second country or region only 90+ data are available, we might ask whether these data still contain enough information to detect mortality deceleration. To get an idea of the sample size that is required to draw reliable conclusions under the given mortality pattern $\varvec{\theta }$, we could determine the sample size $n_{90+}$ of the subset of survivors to ages 90 and above, which satisfies $n_{80+}{\mathcal {I}}_{80+} = n_{90+}{\mathcal {I}}_{90+}$.

Alternatively, sample size considerations could concern the precision of the estimate ${\hat{\sigma }}^2$, which is given by $n_{x+}^{-1}\kappa ^2_{x+}$, if for the (assumed) mortality regime $\varvec{\theta }$, the frailty variance $\sigma ^2$ is sufficiently large to make $\kappa ^2$ the correct variance term (see Sect. 3.3). For a given value of $\kappa ^2_{x+}$, we can either get an initial idea of the precision of ${\hat{\sigma }}^2$ if the size $n_{x+}$ of the $x+$ data of the country is known, or we can determine the minimum sample size $n_{x+}$ that is needed for a desired precision, and see whether potential datasets would fulfill this requirement.

Finally, both the age range of a dataset and its sample size affect the power of the likelihood ratio test to detect mortality deceleration. For a given mortality regime $\varvec{\theta }$, formula (6) allows us to assess what level of power the test will achieve if inference is based on all survivors to ages 90+, or on all survivors to ages 80+.

As all of the above quantities for evaluating the design aspects of the age range and the sample size depend on the true unknown parameter $\varvec{\theta }$, we will present some empirical results for specific scenarios in the next section.

## Empirical results

In this section, we will study the effects of different designs on the information contained in a dataset for some specific scenarios. We assume that *X* follows a gamma-Gompertz distribution and describes lifespan after age 60; that is, $x=0$ corresponds to age 60. We choose three scenarios for $\varvec{\theta }$, all with the same Gompertz parameters, $a=0.015$ and $b=0.085$, but with different values for the frailty variance. Scenario $S_1$ with $\sigma ^2=0.043$ corresponds to the gamma-Gompertz model estimated from the female sample in Sect. 6. For Scenario $S_2$ with $\sigma ^2=0.021$, the frailty variance is roughly halved, representing a less heterogeneous population for which mortality deceleration is less pronounced. In Scenario $S_3$ with $\sigma ^2=0$, there is no mortality deceleration. In Section S.3.3 of the supplementary material, we present results for additional Scenarios $S_4$ to $S_6$ with the same three values for the frailty variance as above, but different values for the Gompertz parameters. In particular, we set $a=0.021$ and $b=0.082$ equal to the estimates obtained from a gamma-Gompertz fit to the male sample in Sect. 6. With regard to the age range, we assess the information measures for complete observations of *X* (that is, ages 60 and above, 60+), as well as for left-truncated observations corresponding to survivors to ages 80 and above (80+), 85+, and 90+. The effect of the sample size will be examined by considering different sizes of the subset of survivors to ages 90+; namely, $n_{90+}=$ 10,000 (small), $n_{90+}=$ 20,000 (medium), or $n_{90+}=$ 105,000 (large). The small and medium sizes are close to the sizes of the male and female samples in Sect. 6. All computations are run in R (R Core Team 2019), and the numerical integration to calculate the Fisher information ${\varvec{I}}(\varvec{\theta })$ is performed using function integrate().

In a preliminary analysis, we assessed the performance of our approach of using numerical integration to calculate ${\varvec{I}}(\varvec{\theta })$. For that purpose, we generated 1,000 samples for each of the scenarios $S_1$ to $S_3$, in which three initial sample sizes at age 60 were determined to yield the desired $n_{90+}$ given above. For each sample, we estimated the parameters of the gamma-Gompertz model based on the full sample (60+), and based on the subsets of survivors to ages 80+, 85+, and 90+, by maximizing the log-likelihoods numerically using function nlm(). We then calculated the averages of the observed Fisher information matrices evaluated at the MLEs across the 1,000 samples of each fixed setting, $\varvec{\bar{{\mathcal {J}}}}=\frac{1}{1000}\sum _{r=1}^{1000}\varvec{{\mathcal {J}}}(\varvec{{\hat{\theta }}_n^{(r)}})$. Finally, these averages were compared to the Fisher information matrices ${\varvec{I}}(\varvec{\theta })$, that were scaled by the theoretical size $n_{\cdot +}$ of a sample from the respective setting. The results are reported in Table S.1 in the supplementary material. As expected, mean relative differences decrease with sample size, width of age range and size of the frailty variance. For ages 60+ and 80+ differences are negligible throughout, and for 85+ surpass 0.02 only in the no-frailty scenario ($n_{90+}=$ 10,000: 0.03389; $n_{90+}=$ 20,000: 0.02114). For ages 90+ and smallest sample size $n_{90+}=$ 10,000 the values are $S_1$: 0.05487, $S_2$: 0.06592 and $S_3$: 0.12065.

### Effect of the age at left truncation

In the following, we quantify how different restrictions of the age range covered by a dataset affect the amount of information that is provided by the data. We mainly focus on the criterion for $D_A$-optimality (see Sect. 3.2), that is, ${\mathcal {I}}=\kappa ^{-2}$.

**Fig. 1:**
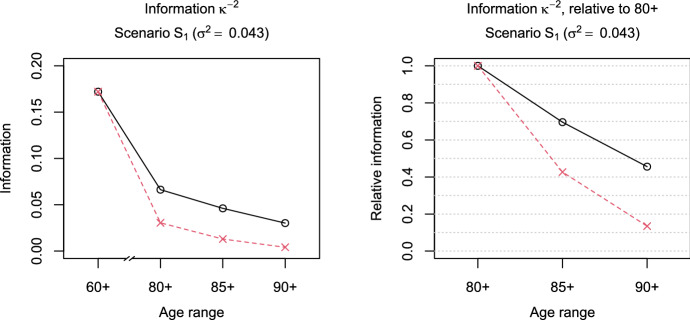
Information measure ${\mathcal {I}}=\kappa ^{-2}$ (black-solid line, circles) and scaled measure ${\mathcal {I}}^{(s)}$ (red-dashed line, crosses) under Scenario $S_1$ depending on the age range of the data (left to right: 60+, 80+, 85+, or 90+). Left: absolute values of (scaled) ${\mathcal {I}}$, right: (scaled) ratios ${\mathcal {I}}_{x+}/{\mathcal {I}}_{80+}$ for $x=80,85,90$ (connection of the values by lines is only for ease of visual inspection) (Color figure online)

**Fig. 2:**
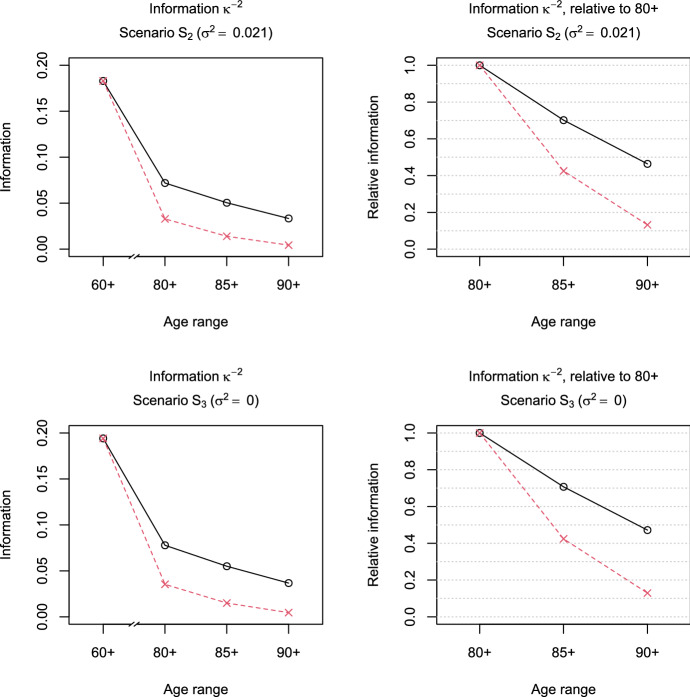
Information measure ${\mathcal {I}}=\kappa ^{-2}$ (black-solid line, circles) and scaled measure ${\mathcal {I}}^{(s)}$ (red-dashed line, crosses) under Scenarios $S_2$ (top) and $S_3$ (bottom) depending on the age range of the data (left to right: 60+, 80+, 85+, or 90+). Left: absolute values of (scaled) ${\mathcal {I}}$, right: (scaled) ratios ${\mathcal {I}}_{x+}/{\mathcal {I}}_{80+}$ for $x=80,85,90$ (Color figure online)

The left panel of Fig. 1 shows the values of ${\mathcal {I}}$ and its scaled version ${\mathcal {I}}^{(s)}$ for Scenario $S_1$ when the observations are complete (60+) or left-truncated at higher ages (80+, 85+, or 90+). We find that the amount of information contained in an observation decreases as the age of left truncation increases. This effect is even more pronounced for the scaled measure of information ${\mathcal {I}}^{(s)}$, because the probability of observing deaths decreases at the higher ages. Hence, in terms of $\kappa ^{-2}$, a situation in which only survivors to ages 90+ can be studied indeed provides less information than a situation in which survivors to ages 80+ can be studied, indicating that mortality deceleration is more difficult to assess for higher ages of left truncation. The right panel of Fig. 1 displays the ratios ${\mathcal {I}}_{x+}/{\mathcal {I}}_{80+}$ and ${\mathcal {I}}^{(s)}_{x+}/{\mathcal {I}}^{(s)}_{80+}$ for $x=80,85,90$ in Scenario $S_1$. We see that if only data on survivors to ages 90+ are available, more than half of the information is lost compared to the situation in which data on survivors to ages 80+ are available. Taking into account the smaller size of the subset of survivors to ages 90+, the loss even amounts to around 87%. The results for Scenarios $S_2$ and $S_3$ are similar (cf. Fig. 2).

We have already briefly discussed in Sect. 3.3 the relationship between the information measure $\kappa ^{-2}$ and the asymptotic variance of the estimator ${\hat{\sigma }}^2$. In settings with sufficiently large $\sigma ^2$, the variance of ${\hat{\sigma }}^2$ is approximately equal to $\kappa ^2$ scaled by the inverse of the sample size. The top-left panel of Figure S.1 verifies this for the medium-sized Scenario $S_1$ with different observation schemes (60+, 80+, 85+, and 90+) by comparing the empirical variance of ${\hat{\sigma }}^2$ across the 1,000 replications with the scaled $\kappa ^2$. In contrast, if $\sigma ^2=0$, the asymptotic variance of ${\hat{\sigma }}^2$ is not given by the scaled $\kappa ^2$, as shown in the bottom-left panel of Figure S.1 for the medium-sized Scenario $S_3$. However, the relative changes in the scaled $\kappa _{x+}^2$ across different age ranges $x+$ are in line with the relative changes in the empirical variances for both Scenario $S_1$ (top-right panel of Figure S.1) and Scenario $S_3$ (bottom-right panel of Figure S.1). Consequently, ratios ${\mathcal {I}}^{(s)}_{x+}/{\mathcal {I}}^{(s)}_{y+}$ can be readily interpreted in terms of information gain or variance reduction when considering different age ranges $x+$ and $y+$, even if $\sigma ^2=0$. For example, in Scenario $S_3$, about 82% of the information in the full sample 60+ is lost if only survivors to ages 80+ can be studied.

While the importance of the variance parameter $\sigma ^2$ for the assessment of mortality deceleration suggested that we should use the criterion of $D_A$-optimality, we also looked into the performance of the other information measures introduced in Sect. 2.3. The results for Scenarios $S_1$ and $S_3$ are presented in Figures S.2 and S.3 in the supplementary material. The absolute values of the information measures and their patterns across the different age ranges for *A*- and *E*-optimality are very close to the ones we observed for $D_A$-optimality with ${\mathcal {I}}=\kappa ^{-2}$. This can be explained by the fact that the information measure for *A*-optimality, that is, the inverse of the sum of the eigenvalues of $[{\varvec{I}}(\varvec{\theta })]^{-1}$, is dominated by one eigenvalue of relatively large magnitude in the current setting. As this largest eigenvalue of the inverse information matrix is at the same time the target of the criterion for *E*-optimality and in this case also closely related to the measure $\kappa ^2$ for $D_A$-optimality, the three criteria yield very similar results. In contrast, the criterion of *D*-optimality suggests somewhat larger relative losses in information when restricting the age range. This is a consequence of directly accounting for the increased uncertainty in all directions of the parameter space, as the eigenvalues of ${\varvec{I}}(\varvec{\theta })$ are multiplied in the measure $\mathrm {det}({\varvec{I}}(\varvec{\theta }))$.

**Fig. 3:**
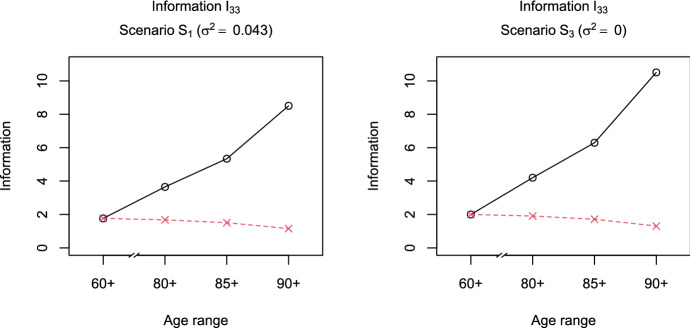
Information measure ${\mathcal {I}}=[{\varvec{I}}(\varvec{\theta })]_{33}$ (black-solid line, circles) and scaled measure ${\mathcal {I}}^{(s)}$ (red-dashed line, crosses) depending on the age range of the data (left to right: 60+, 80+, 85+, or 90+) under Scenarios $S_1$ (left) and $S_3$ (right) (Color figure online)

The quantity $\kappa ^{-2}$, considered for $D_A$-optimality, measures the information contained in an observation about the parameter $\sigma ^2$, and takes into account the correlation between ${\hat{\sigma }}^2$ and the estimates of the Gompertz parameters ${{\hat{a}}}$ and ${{\hat{b}}}$. Alternatively, we could study the information measure $[{\varvec{I}}(\varvec{\theta })]_{33}$ given by

$$\begin{aligned} {[}{\varvec{I}}(\varvec{\theta })]_{33}=-{\mathbb {E}}\left[ \frac{\partial ^2}{\partial \left( \sigma ^2\right) ^2}\ln {f_X(X;\varvec{\theta })}\right] . \end{aligned}$$

This element of the Fisher information matrix describes the average curvature of the log-density of the gamma-Gompertz model with respect to $\sigma ^2$ for fixed Gompertz parameters *a* and *b*. Figure 3 shows that in terms of this measure, the information increases with the increasing age of left truncation in Scenarios $S_1$ and $S_3$. This supports the idea that observations of later ages at death carry more information about the potential deceleration, as measured by $\sigma ^2$. However, looking at the scaled measure reveals that, in practice, this effect is compensated for by the decreasing number of survivors to higher ages.

The above findings regarding the effect of the age at left truncation on the different information measures generally hold also for the Scenarios $S_4$ and $S_6$ with modified Gompertz parameters (see Figures S.4 to S.7 in the supplementary material). Increases in the age at left truncation result in considerable information loss according to all the criteria, with the exception of ${\mathcal {I}}=[{\varvec{I}}(\varvec{\theta })]_{33}$, for which the information loss is only revealed if the smaller size of the subset of survivors to higher ages is taken into account. Compared to Scenarios $S_1$ and $S_3$, the higher initial level of mortality *a* in Scenarios $S_4$ and $S_6$ leads to smaller absolute values of the information measures and slightly larger information losses when restricting the age range. This is expected because the higher initial mortality leads to stronger selection effects and thus, a stronger decrease in the variance of frailty among survivors to higher ages, as well as to lower probabilities of surviving to these ages.

Finally, the information measures computed in this subsection refer to single observations, such that the derived conclusions about the effects of the age at left truncation should be valid for samples of any reasonable size under the different scenarios. Further aspects regarding the size of datasets under study are discussed in the following.

### Sample size considerations

Let us suppose that old-age mortality is studied in countries that experience the same or very similar mortality patterns. It is clear that the different population sizes affect the assessment of mortality deceleration in the different countries. If the data for the different countries also cover different age ranges, then we want to know, for example, what size $n_{90+}$ a subset of survivors to ages 90+ would have to be to carry as much information as a subset of survivors to ages 80+ from a different country; that is, $n_{80+}{\mathcal {I}}_{80+} = n_{90+}{\mathcal {I}}_{90+}$. In Scenarios $S_1$ to $S_3$, the ratios ${\mathcal {I}}_{80+}/{\mathcal {I}}_{90+}$ for ${\mathcal {I}}=\kappa ^{-2}$ are 2.194, 2.156, and 2.120, respectively. Thus, a sample of survivors to ages 90+ needs to be more than twice as large as a sample of survivors to ages 80+ in order to provide the same amount of information. Moreover, the ratios are increasing in the level of heterogeneity $\sigma ^2$. For Scenarios $S_4$ to $S_6$, we obtain the slightly larger ratios 2.322, 2.278, and 2.237, reflecting the stronger selection effects in these settings.

If the underlying $\sigma ^2$ is sufficiently large, we can use the measure $\kappa ^2$ to draw conclusions about the precision of ${\hat{\sigma }}^2$ for specific sample sizes. In Scenario $S_1$, if the sample of survivors to ages 90+ consists of about $n_{90+}=$ 20,000 individuals, this would yield a precision of the estimated frailty variance of about $\mathrm {var}({\hat{\sigma }}^2)\approx n^{-1}_{90+}\kappa ^2_{90+}=0.00165$.

### Power of the likelihood ratio test

We now evaluate how the age range and the sample size of a dataset affect the performance of the likelihood ratio test for assessing mortality deceleration in the gamma-Gompertz model. The power of the test to detect a positive $\sigma ^2$ in Scenarios $S_1$ and $S_2$ based on different subsets and sample sizes is calculated from (6) at a level of $\alpha =0.05$, and is reported in Table 1. As expected, we find that the power of the test increases if the sample size increases, if the age at left truncation decreases, and if the frailty variance is larger. In both scenarios, the power to detect mortality deceleration decreases by more than two-thirds if inference is based only on survivors to ages 90+, rather than on all survivors to ages 80+ for samples of medium size.

**Table 1:** Power $\beta$ of the likelihood ratio test, performed at the $5\%$ level, according to formula (6), under Scenarios $S_1$ ($\sigma ^2=0.043$) and $S_2$ ($\sigma ^2=0.021$) for three sample size settings (s—small, m—medium, l—large) and varying age range

Scen.	*n*	Survivors to ages							
		$60+$		$80+$		$85+$		$90+$	
		$n_{60+}$	$\beta _{60+}$	$n_{80+}$	$\beta _{80+}$	$n_{85+}$	$\beta _{85+}$	$n_{90+}$	$\beta _{90+}$
$S_1$	s	73,558	0.999	33,841	0.653	20,740	0.377	10,000	0.185
	m	147,116	1.000	67,681	0.892	41,480	0.593	20,000	0.278
	l	772,361	1.000	355,327	1.000	217,771	0.996	105,000	0.782
$S_2$	s	76,853	0.801	35,123	0.278	21,290	0.169	10,000	0.104
	m	153,706	0.970	70,245	0.440	42,581	0.251	20,000	0.135
	l	806,956	1.000	368,788	0.962	223,548	0.721	105,000	0.344

The performance of the likelihood ratio test under the Scenarios $S_4$ and $S_5$, with modified Gompertz parameters, is documented in Table S.2 in the supplementary material and leads to the same conclusions as above. In addition, we see that in Scenario $S_4$ the test has lower power to detect the positive $\sigma ^2$ based on the survivors to ages 85+ or 90+ than in the corresponding Scenario $S_1$. This is due to the higher initial level of mortality *a* in $S_4$ which reduces the heterogeneity in the mortality risks at the higher ages. The power of the test based on survivors to ages 60+ and 80+ in Scenarios $S_4$ and $S_5$ is not directly comparable to the power in the corresponding Scenarios $S_1$ and $S_2$, because of the different sizes of the subsets 60+ and 80+ under the two settings for the Gompertz parameters.

Apart from calculating power values for given parameter configurations, formula (6) provides a tool for determining what age range a dataset should cover to ensure that the likelihood ratio test will achieve a certain level of power. From Table 1, we see that for the medium-sized Scenario $S_1$, the likelihood ratio test will detect mortality deceleration in about 89.2% of cases based on the sample of survivors to ages 80+. As for any left-truncation age *x*, the size of the sample $x+$ can be calculated based on the given (or estimated) gamma-Gompertz parameters and a known subset size (e.g., here, $n_{90+}=$ 20,000), we can determine the left-truncation age *x* such that the power is increased to 95%. For the medium-sized Scenario $S_1$, we need to include all survivors to ages 78 and above for the test to reach a power of 95%.

The above power calculations are based on relatively large sample sizes as motivated by real cohort data. We have seen that the assessment of mortality deceleration can be demanding even based on datasets of such size especially in case of restricted age ranges. Smaller sample sizes will lower the power of the test to detect a positive frailty variance also for the subsets of survivors to ages 60+ or 80+. A larger underlying frailty variance leads to stronger deceleration in the hazard rate and will generally be favorable for detecting the phenomenon. However, smaller sample sizes and thus increased uncertainty about the parameters can counteract this effect, as can be seen from formula (6). While the formula provides only a large-sample approximation to the power of the likelihood ratio test, the sample sizes in human mortality studies are expected to be generally large enough for the approximation to yield valid results.

## A study on old-age mortality among French-Canadians

In this section, we apply the proposed methods for evaluating study design and deriving design recommendations to a study on old-age mortality among Catholic French-Canadians born at the end of the nineteenth century.

The dataset contains information on 20,917 females and 10,878 males who were born in the Province of Quebec between 1880 and 1896, and who died in Quebec at ages 90 and above between 1970 and 2009. To validate the individual exact survival times, birth registration documents and death certificates from Quebec’s parish register archives were linked. Further details on the data and the validation procedure can be found in Ouellette and Bourbeau (2014) and Ouellette (2016), who studied earlier versions of this dataset that covered only the centenarians, that is, survivors to ages 100 and above.

**Fig. 4:**
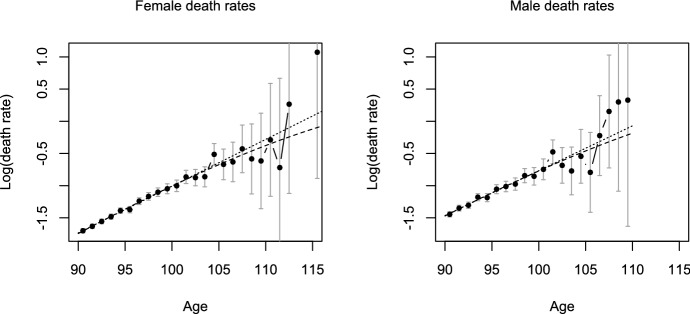
Death rates of French-Canadian females (left) and males (right): empirical death rates (solid line, circles) with 95%-confidence intervals (gray), gamma-Gompertz fit (dashed) and Gompertz fit (dotted) (Color figure online)

The analysis of the French-Canadian mortality data based on the gamma-Gompertz model is conducted separately for the female and the male sample. The starting age of the model is assumed to be 60, and the likelihood is adapted for the left truncation at age 90. We obtain estimates of the frailty variance of ${\hat{\sigma }}^2=0.043$ for the females and ${\hat{\sigma }}^2=0.037$ for the males. The likelihood ratio test for $H_0\!: \sigma ^2=0$ leads to *p*-values of 0.121 for the females and 0.283 for the males, indicating that the data do not provide much evidence against the null hypothesis of no mortality deceleration. These findings are in contrast to those for the fitted hazards and the empirical death rates, which are displayed in Fig. 4, and suggest a deceleration, at least for the females. Indeed, it turns out that the likelihood ratio test has relatively low power to detect mortality deceleration in the given settings. According to formula (6) with the estimated values of the parameters, the power of the likelihood ratio test at the 5% level based on a 90+ sample of the given size is $28.7\%$ in the female setting and $14.2\%$ in the male setting, respectively. Therefore, we want to investigate how a further extension of the dataset that would include deaths at earlier ages—say, between ages 85 and 89, or between 80 and 89—could impact the assessment of mortality deceleration.

**Fig. 5:**
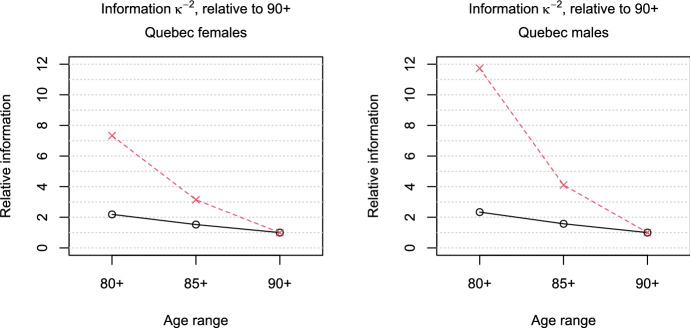
Ratios ${\mathcal {I}}_{x+}/{\mathcal {I}}_{90+}$ (black-solid line, circles) or ${\mathcal {I}}^{(s)}_{x+}/{\mathcal {I}}^{(s)}_{90+}$ (red-dashed line, crosses) for the information measure ${\mathcal {I}}=\kappa ^{-2}$ depending on the age of left truncation $x=80,85,90$, under the parameter settings estimated from the samples of French-Canadian females (left) and males (right)

First, we examine the behavior of the information measure ${\mathcal {I}}=\kappa ^{-2}$ for observations from a gamma-Gompertz model with parameter values equal to the estimates obtained from the female or the male sample, respectively. Figure 5 depicts the ratios ${\mathcal {I}}_{x+}/{\mathcal {I}}_{90+}$ for left truncation at the ages $x=80,85,90$, as well as the ratios of the scaled measure ${\mathcal {I}}^{(s)}$. In both the female and the male setting, an observation left-truncated at age 80 would be more than twice as informative as an observation left-truncated at age 90. Taking into account the increasing number of observations when younger ages at death are included by looking at ${\mathcal {I}}^{(s)}$, observations left-truncated at age 85 are already more than twice as informative as observations left-truncated at age 90. In other words, the extended dataset that includes all survivors to ages 85 and above would contain more than twice as much information as the current dataset of survivors to ages 90 and above. Indeed, compared to the 90+ sample, the female 85+ sample would provide about three times as much information, and the male 85+ sample would provide about four times as much information.

**Table 2:** Power $\beta$ of the likelihood ratio test, performed at the $5\%$ level, according to formula (6), under the parameter settings estimated from the French-Canadian data for different age ranges $x+$ and resulting sample sizes $n_{x+}$

Scenario	Survivors to ages					
	$80+$		$85+$		$90+$	
	$n_{80+}$	$\beta _{80+}$	$n_{85+}$	$\beta _{85+}$	$n_{90+}$	$\beta _{90+}$
Females	70,085	0.901	43,126	0.609	20,917	0.287
Males	54,577	0.627	28,369	0.316	10,878	0.142

Second, Table 2 summarizes the effects of expanding the age range of the current dataset on the power of the likelihood ratio test which is performed at a level of $5\%$. The calculations are again based on formula (6), with the estimates obtained from the female and the male samples, respectively, inserted for the parameter values. The sizes of the expanded 85+ and 80+ datasets are computed from the known size of the population of survivors to ages 90+ and the fitted gamma-Gompertz model. For both the female and the male data, we find that expanding the dataset to deaths between ages 85 and 89 would more than double the power of the likelihood ratio test. For the female setting, the power of the test based on the 85+ sample is around $60.9\%$. In line with the considerations in Sect. 5.3, the age of left truncation that is required in this setting to achieve the desired power level of $80\%$ is found to be age 82.

In conclusion, our results show that the failure of the likelihood ratio test to reject the hypothesis of no mortality deceleration in the female 90+ sample can be explained to some extent by the low power of the test in the specific setting. Both the proposed information measure $\kappa ^{-2}$ and the power calculations for the likelihood ratio test demonstrate that an expansion of the existing dataset on French-Canadian mortality could greatly improve the assessment of mortality deceleration in this population. In practice, these potential improvements have to be weighed against the costs of collecting—and validating—the additional data.

## Discussion

We have investigated the use of Fisher information-based criteria for planning and evaluating studies that assess mortality deceleration in the framework of the gamma-Gompertz model. Our aim was to derive recommendations for settings in which the parameters of interest could be reliably estimated, and a deceleration in the death rates could be detected with a high probability. As validation of the ages at death is often required in old-age mortality studies, these settings are characterized by the age range covered by the data and the sample size.

The essential component of the proposed methods is the computation of the Fisher information matrix for potentially left-truncated observations from a gamma-Gompertz model. Due to a lack of closed-form expressions, the information matrix is obtained using numerical integration of analytically determined second-order partial derivatives of the log-density. Different criteria for evaluating study designs can be derived from the Fisher information. Given the importance of the frailty variance parameter in assessing mortality deceleration in the gamma-Gompertz model, we focus primarily on a criterion of $D_A$-optimality, whereby the Gompertz parameters are treated as nuisance. The resulting measure of information is the reciprocal of the element of the inverse Fisher information that corresponds to $\sigma ^2$. It allows us to quantify the effects of the sample size and the age range covered by a dataset on the amount of information that this dataset contains about $\sigma ^2$. Based on the computation of the Fisher information matrix, we are also able to calculate the power of the likelihood ratio test to detect mortality deceleration in specific scenarios. As a result, recommendations can be given about what age range a dataset needs to cover for the likelihood ratio test to achieve a certain power. In the illustration with a study on French-Canadian mortality, the information measures and the power calculations clearly demonstrate that the assessment of mortality deceleration could be greatly improved if the current dataset, which includes only survivors to ages 90 and above, was extended to also include deaths at the earlier ages 85–89.

Here, we only consider changes in the age at left truncation (the age range covered by the data) that apply to all survivors in a cohort, which is the most common setting in demographic studies. We could, however, extend these considerations to more complex design questions for which actually random samples could be drawn from the observed survivors to particular ages. In such situations, the quantification of information in particular observations would be even more crucial for attaining an optimal design. However, addressing such questions is beyond the scope of the current research.

The present work has some limitations. As our focus is on the assessment of mortality deceleration—the deviation from the log-linear hazard trajectory of the Gompertz model at high ages—, we have stayed within the framework of the gamma-Gompertz model here. However, as the concepts of the Fisher information and of optimal design are defined for any parametric model, the proposed methods should be applicable in a wider context.

When the age range can be extended to appreciably lower ages the exponential increase of senescent mortality may no longer hold and questions of model choice for the baseline hazard arise. Established model selection techniques can help here (see Burnham and Anderson 2002). In such cases most problems discussed in this paper would be obsolete though. It should be noted, however, that also commonly used model choice criteria, such as Akaike’s Information Criterion (AIC), are affected by non-standard conditions induced by a boundary parameter (see Böhnstedt and Gampe 2019).

Although the approach of using numerical integration to compute the Fisher information matrix in the gamma-Gompertz model seems to perform well, it prevents us from deriving general analytical formulas that describe the effects of the sample size and the age range of a dataset. Nonetheless, empirical studies for specific parameter settings and design choices as presented here can serve as a basis for formulating recommendations.

In this context, we have to bear in mind that the gamma-Gompertz model provides a non-standard setting, and, hence, that the information measures are not directly related to estimator variability in the boundary case ($\sigma ^2=0$). We have, however, shown that comparative statements on information gain or variance reduction are still meaningful when designs covering different age ranges are compared.

Finally, as the assessment of the performance of different study designs for the gamma-Gompertz model based on the Fisher information depends on the true underlying parameter values, it is valid only locally. Still, the robustness of the design’s performance can be checked by evaluating the information measures using a range of possible parameter values.

## Supplementary Information

Below is the link to the electronic supplementary material.

Supplementary material 1 (pdf 745 KB)

## Data Availability

The dataset on the French-Canadian mortality analyzed in the present manuscript cannot be made available for reasons of confidentiality.

## Code availability

R-code to reproduce the results of Sect. 5 is available from the corresponding author upon request.
