# Supplementary material for: Information measures and design issues in the study of mortality deceleration: findings for the gamma-Gompertz model
Source: Lifetime Data Anal. 2021 Feb 25;27(3):333–56. doi: 10.1007/s10985-021-09518-4 (PMC8238756; doi:10.1007/s10985-021-09518-4)
Supplement: Supplementary file 1 — Supplementary material 1 (pdf 745 KB) [file 10985_2021_9518_MOESM1_ESM.pdf]

Supplementary Material for

Information measures and design issues in the study of mortality deceleration: Findings for the gamma-Gompertz model

Marie Böhnstedt*[,1,2], Jutta Gampe[1], and Hein Putter[2]

[1] Max Planck Institute for Demographic Research, Rostock, Germany

[2] Department of Biomedical Data Sciences, Leiden University Medical Center, Leiden, The Netherlands

December 2020

Contents

S.1 Derivatives of gamma-Gompertz log-densities **1**

S.2 Computational details **4**

S.3 Additional figures and tables for empirical studies **5**

S.1 Derivatives of gamma-Gompertz log-densities

In the following subsections, we give explicit formulas of the second-order partial derivatives of the log-density of complete or left-truncated observations from a gamma-Gompertz model. These derivatives are the basis for computing the Fisher information matrix $\boldsymbol{I}(\boldsymbol{\theta})$ according to formula (3) or (3') in the main paper,

$$\boldsymbol{I}(\boldsymbol{\theta}) = -\mathbb{E}\left[\frac{\partial^2}{\partial\boldsymbol{\theta}\partial\boldsymbol{\theta}^T} \ln f_X(X;\boldsymbol{\theta})\right]. \tag{3}$$

*e-mail: boehnstedt@demogr.mpg.de

S.1.1 Complete Data

For the gamma-Gompertz model (1), the log-density $\ln f_X(\cdot; a, b, \sigma^2)$ of complete data X takes the form

$$\ln f_X(x; a, b, \sigma^2) = \ln a + bx - \left(1 + \frac{1}{\sigma^2}\right) \ln \left[1 + \sigma^2 \frac{a}{b} \left(e^{bx} - 1\right)\right].$$

Its partial derivatives with respect to the parameters are calculated as

$$\begin{aligned}
\frac{\partial \ln f_X}{\partial a} &= \frac{1}{a} - \frac{(\sigma^2+1)}{b} \cdot \frac{e^{bx} - 1}{1 + \sigma^2 \frac{a}{b}(e^{bx} - 1)}, \\
\frac{\partial \ln f_X}{\partial b} &= x - \frac{a(\sigma^2+1)}{b^2} \cdot \frac{bxe^{bx} - (e^{bx} - 1)}{1 + \sigma^2 \frac{a}{b}(e^{bx} - 1)}, \quad \text{and} \\
\frac{\partial \ln f_X}{\partial \sigma^2} &= \frac{1}{\sigma^4} \ln \left[1 + \sigma^2 \frac{a}{b}\left(e^{bx} - 1\right)\right] - \left(1 + \frac{1}{\sigma^2}\right) \frac{a}{b} \cdot \frac{e^{bx} - 1}{1 + \sigma^2 \frac{a}{b}(e^{bx} - 1)}.
\end{aligned}$$

The second-order partial derivatives equal

$$\begin{aligned}
\frac{\partial^2 \ln f_X}{\partial a^2} &= -\frac{1}{a^2} + \frac{\sigma^2(\sigma^2+1)}{b^2} \cdot \frac{(e^{bx} - 1)^2}{[1 + \sigma^2 \frac{a}{b}(e^{bx} - 1)]^2} \\
\frac{\partial^2 \ln f_X}{\partial a \partial b} &= \frac{(\sigma^2+1)}{b^2} \cdot \frac{(e^{bx} - 1) - bxe^{bx}}{[1 + \sigma^2 \frac{a}{b}(e^{bx} - 1)]^2} \\
\frac{\partial^2 \ln f_X}{\partial a \partial \sigma^2} &= \frac{1}{b^2} \cdot \frac{a(e^{bx} - 1)^2 - b(e^{bx} - 1)}{[1 + \sigma^2 \frac{a}{b}(e^{bx} - 1)]^2} \\
\frac{\partial^2 \ln f_X}{\partial b^2} &= \frac{a(\sigma^2+1)}{b} \cdot \frac{\frac{2}{b}xe^{bx} - \frac{2}{b^2}(e^{bx} - 1) - \sigma^2 \frac{a}{b^3}(e^{bx} - 1)^2 + (\sigma^2 \frac{a}{b} - 1)x^2 e^{bx}}{[1 + \sigma^2 \frac{a}{b}(e^{bx} - 1)]^2} \\
\frac{\partial^2 \ln f_X}{\partial b \partial \sigma^2} &= \frac{a}{b} \cdot \frac{[\frac{1}{b}(e^{bx} - 1) - xe^{bx}][1 - \frac{a}{b}(e^{bx} - 1)]}{[1 + \sigma^2 \frac{a}{b}(e^{bx} - 1)]^2} \\
\frac{\partial^2 \ln f_X}{\partial (\sigma^2)^2} &= -\frac{2}{\sigma^6} \ln \left[1 + \sigma^2 \frac{a}{b}\left(e^{bx} - 1\right)\right] + \frac{2}{\sigma^4} \frac{a}{b} \cdot \frac{e^{bx} - 1}{1 + \sigma^2 \frac{a}{b}(e^{bx} - 1)} \\
&\quad + \left(1 + \frac{1}{\sigma^2}\right) \frac{a^2}{b^2} \cdot \frac{(e^{bx} - 1)^2}{[1 + \sigma^2 \frac{a}{b}(e^{bx} - 1)]^2}. \qquad \text{(S.1)}
\end{aligned}$$

In the limit $\sigma^2 \to 0$, we obtain

$$\begin{aligned}
\frac{\partial^2 \ln f_X}{\partial a^2} &= -\frac{1}{a^2} \\
\frac{\partial^2 \ln f_X}{\partial a \partial b} &= \frac{1}{b^2}(e^{bx} - 1) - \frac{1}{b} xe^{bx}
\end{aligned}$$

$$\begin{aligned}
\frac{\partial^2 \ln f_X}{\partial a \partial \sigma^2} &= \frac{a}{b^2}(e^{bx}-1)^2 - \frac{1}{b}(e^{bx}-1) \\
\frac{\partial^2 \ln f_X}{\partial b^2} &= \frac{2a}{b^2} x e^{bx} - \frac{2a}{b^3}(e^{bx}-1) - \frac{a}{b} x^2 e^{bx} \\
\frac{\partial^2 \ln f_X}{\partial b \partial \sigma^2} &= \frac{a}{b}\left[\frac{1}{b}(e^{bx}-1) - x e^{bx}\right]\left[1 - \frac{a}{b}(e^{bx}-1)\right] \\
\frac{\partial^2 \ln f_X}{\partial (\sigma^2)^2} &= -\frac{2a^3}{3b^3}(e^{bx}-1)^3 + \frac{a^2}{b^2}(e^{bx}-1)^2,
\end{aligned}$$

where we have applied the rule of L'Hôpital for the last equation.

S.1.2 Left-Truncated Data

For the gamma-Gompertz model (1), the log-density $\ln f_{X|X>y}(\cdot; a, b, \sigma^2)$ for data $(X \mid X > y)$, left-truncated at age y, takes the form

$$\begin{aligned}
\ln f_{X|X>y}(x; a, b, \sigma^2) &= \ln f_X(x; a, b, \sigma^2) - \ln S_X(y; a, b, \sigma^2) \\
&= \ln f_X(x; a, b, \sigma^2) + \frac{1}{\sigma^2} \ln\left[1 + \sigma^2 \frac{a}{b}(e^{by}-1)\right],
\end{aligned}$$

for $x > y$. The partial derivatives of the first summand have already been presented in Section S.1.1. Thus, we focus on the second summand here, which we denote as $g(y; a, b, \sigma^2)$. The partial derivatives of g with respect to the parameters are computed as

$$\begin{aligned}
\frac{\partial g}{\partial a} &= \frac{1}{b} \cdot \frac{e^{by}-1}{1 + \sigma^2 \frac{a}{b}(e^{by}-1)}, \\
\frac{\partial g}{\partial b} &= \frac{a}{b^2} \cdot \frac{b y e^{by} - (e^{by}-1)}{1 + \sigma^2 \frac{a}{b}(e^{by}-1)}, \qquad \text{and} \\
\frac{\partial g}{\partial \sigma^2} &= -\frac{1}{\sigma^4} \ln\left[1 + \sigma^2 \frac{a}{b}\left(e^{by}-1\right)\right] + \frac{a}{b\sigma^2} \cdot \frac{(e^{by}-1)}{1 + \sigma^2 \frac{a}{b}(e^{by}-1)}.
\end{aligned}$$

The second-order partial derivatives read

$$\begin{aligned}
\frac{\partial^2 g}{\partial a^2} &= -\frac{\sigma^2}{b^2} \cdot \frac{(e^{by}-1)^2}{[1 + \sigma^2 \frac{a}{b}(e^{by}-1)]^2} \\
\frac{\partial^2 g}{\partial a \partial b} &= \frac{1}{b^2} \cdot \frac{1 - e^{by} + b y e^{by}}{[1 + \sigma^2 \frac{a}{b}(e^{by}-1)]^2} \\
\frac{\partial^2 g}{\partial a \partial \sigma^2} &= -\frac{a}{b^2} \cdot \frac{(e^{by}-1)^2}{[1 + \sigma^2 \frac{a}{b}(e^{by}-1)]^2}
\end{aligned}$$

$$
\begin{aligned}
\frac{\partial^2 g}{\partial b^2} &= \frac{a}{b} \cdot \frac{\frac{2}{b^2}(e^{by}-1) + \sigma^2 \frac{a}{b^3}(e^{by}-1)^2 - \frac{2}{b} y e^{by} + (1 - \sigma^2 \frac{a}{b}) y^2 e^{by}}{[1 + \sigma^2 \frac{a}{b}(e^{by}-1)]^2} \\
\frac{\partial^2 g}{\partial b \partial \sigma^2} &= -\frac{a^2}{b^3} \cdot \frac{b y e^{by}(e^{by}-1) - (e^{by}-1)^2}{[1 + \sigma^2 \frac{a}{b}(e^{by}-1)]^2} \\
\frac{\partial^2 g}{\partial (\sigma^2)^2} &= \frac{2}{\sigma^6} \ln \left[1 + \sigma^2 \frac{a}{b}(e^{by}-1)\right] - \frac{2}{\sigma^4} \cdot \frac{\frac{a}{b}(e^{by}-1)}{1 + \sigma^2 \frac{a}{b}(e^{by}-1)} \\
&\quad - \frac{1}{\sigma^2} \left[\frac{\frac{a}{b}(e^{by}-1)}{1 + \sigma^2 \frac{a}{b}(e^{by}-1)}\right]^2 . \qquad \text{(S.2)}
\end{aligned}
$$

In the limit $\sigma^2 \to 0$, we have

$$
\begin{aligned}
\frac{\partial^2 g}{\partial a^2} &= 0 \\
\frac{\partial^2 g}{\partial a \partial b} &= \frac{1}{b^2} \cdot [1 - e^{by} + b y e^{by}] \\
\frac{\partial^2 g}{\partial a \partial \sigma^2} &= -\frac{a}{b^2}(e^{by}-1)^2 \\
\frac{\partial^2 g}{\partial b^2} &= \frac{a}{b}\left[\frac{2}{b^2}(e^{by}-1) - \frac{2}{b} y e^{by} + y^2 e^{by}\right] \\
\frac{\partial^2 g}{\partial b \partial \sigma^2} &= -\frac{a^2}{b^3}\left[b y e^{by}(e^{by}-1) - (e^{by}-1)^2\right] \\
\frac{\partial^2 g}{\partial (\sigma^2)^2} &= \frac{2a^3}{3b^3}(e^{by}-1)^3,
\end{aligned}
$$

by again applying the rule of L'Hôpital for the last equation.

S.2 Computational details on the calculation of the observed Fisher information matrix

The calculation of the observed Fisher information matrix $\boldsymbol{\mathcal{J}}(\hat{\boldsymbol{\theta}}_{\boldsymbol{n}})$ in the gamma-Gompertz model is based on the negative second-order partial derivatives of the log-likelihood and the maximum likelihood estimate (MLE) $\hat{\boldsymbol{\theta}}_{\boldsymbol{n}}$ of the parameter vector $\boldsymbol{\theta} = (a, b, \sigma^2)^T$.

The MLE can be determined by numerical optimization of the log-likelihood using function `nlm()` in R. Optimization over the log-scale of the parameters ensures non-negativity of the parameter estimates. The numerical stability of the estimation problem for values of σ^2 close to zero can be improved by providing also the analytic gradient of the log-likelihood to the optimization routine as well as by using Taylor

expansions of the log-likelihood and the gradient if the current value of σ^2 is smaller than 10^{-5}. In addition, a number of different starting values for the parameter σ^2 should be considered.

Although we have derived explicit formulas for the partial derivatives of the log-density of the gamma-Gompertz model, it turns out that the expressions for the second-order partial derivatives with respect to σ^2, given in (S.1) and (S.2), are not numerically stable if σ^2 approaches zero. Therefore, when calculating $\boldsymbol{\mathcal{J}}(\hat{\boldsymbol{\theta}}_{\boldsymbol{n}})$, we approximate the term $\ln\left[1 + \sigma^2 \frac{a}{b}(e^{bx} - 1)\right]$ in expressions (S.1) and (S.2) by a Taylor expansion if $\hat{\sigma}^2 < 10^{-5}$.

S.3 Additional figures and tables for empirical studies

In this section, we present additional figures and tables displaying some results of our empirical studies in Section 5 of the main paper.

- Table S.1 reports on the performance of the numerical integration approach for computing the Fisher information $\boldsymbol{I}(\boldsymbol{\theta})$.
- The relation between the information measure κ^{-2} and the variance of $\hat{\sigma}^2$, as discussed in Section 3.3 of the main paper, is illustrated in Figure S.1.
- The information measures corresponding to the criteria of D-, A-, and E-optimality (see Section 2.3 of the main paper) are examined in Figures S.2 and S.3 for Scenarios S_1 and S_3, respectively.
- In Section S.3.3, we study the various information measures and the performance of the likelihood ratio test for scenarios with different values for the Gompertz parameters. More precisely, for Scenarios S_4 to S_6, we set $a = 0.021$ and $b = 0.082$, while the values for the frailty variance are the same as in the previous scenarios, that is, $\sigma^2 = 0.043$ in Scenario S_4, $\sigma^2 = 0.021$ in Scenario S_5, and $\sigma^2 = 0$ in Scenario S_6.
  Figure S.4 depicts the patterns of the criterion of D_A-optimality across different age ranges for Scenarios S_4 and S_6, while in Figures S.5 and S.6 the criteria of D-, A-, and E-optimality are presented. Figure S.7 displays the criterion $[\boldsymbol{I}(\boldsymbol{\theta})]_{33}$ for Scenarios S_4 and S_6.
  Finally, the power of the likelihood ratio test to detect a positive σ^2 in Scenarios S_4 ($\sigma^2 = 0.043$) and S_5 ($\sigma^2 = 0.021$) based on different age ranges and

sample sizes at a level of $\alpha = 0.05$ was calculated based on formula (6) of the main paper. The results are presented in Table S.2.

Table S.1: Mean relative difference between the Fisher information $\boldsymbol{I}_{\boldsymbol{n}}(\boldsymbol{\theta})$ and the average $\bar{\boldsymbol{\mathcal{J}}}$ of observed Fisher information matrices across 1,000 replications of Scenarios S_1, S_2, and S_3 for different sample sizes and age ranges

		Survivors to ages			
Scenario	n_{90+}	60+	80+	85+	90+
S_1: $\sigma^2 = 0.043$	10,000	0.00027	0.00344	0.01116	0.05487
	20,000	0.00019	0.00114	0.00512	0.02553
	105,000	0.00009	0.00025	0.00136	0.00579
S_2: $\sigma^2 = 0.021$	10,000	0.00053	0.00356	0.01382	0.06592
	20,000	0.00023	0.00134	0.00614	0.03601
	105,000	0.00009	0.00033	0.00119	0.00591
S_3: $\sigma^2 = 0$	10,000	0.00816	0.01540	0.03389	0.12065
	20,000	0.00611	0.01073	0.02114	0.07251
	105,000	0.00249	0.00504	0.00997	0.02829

S.3.1 Relation between information measures and estimator precision

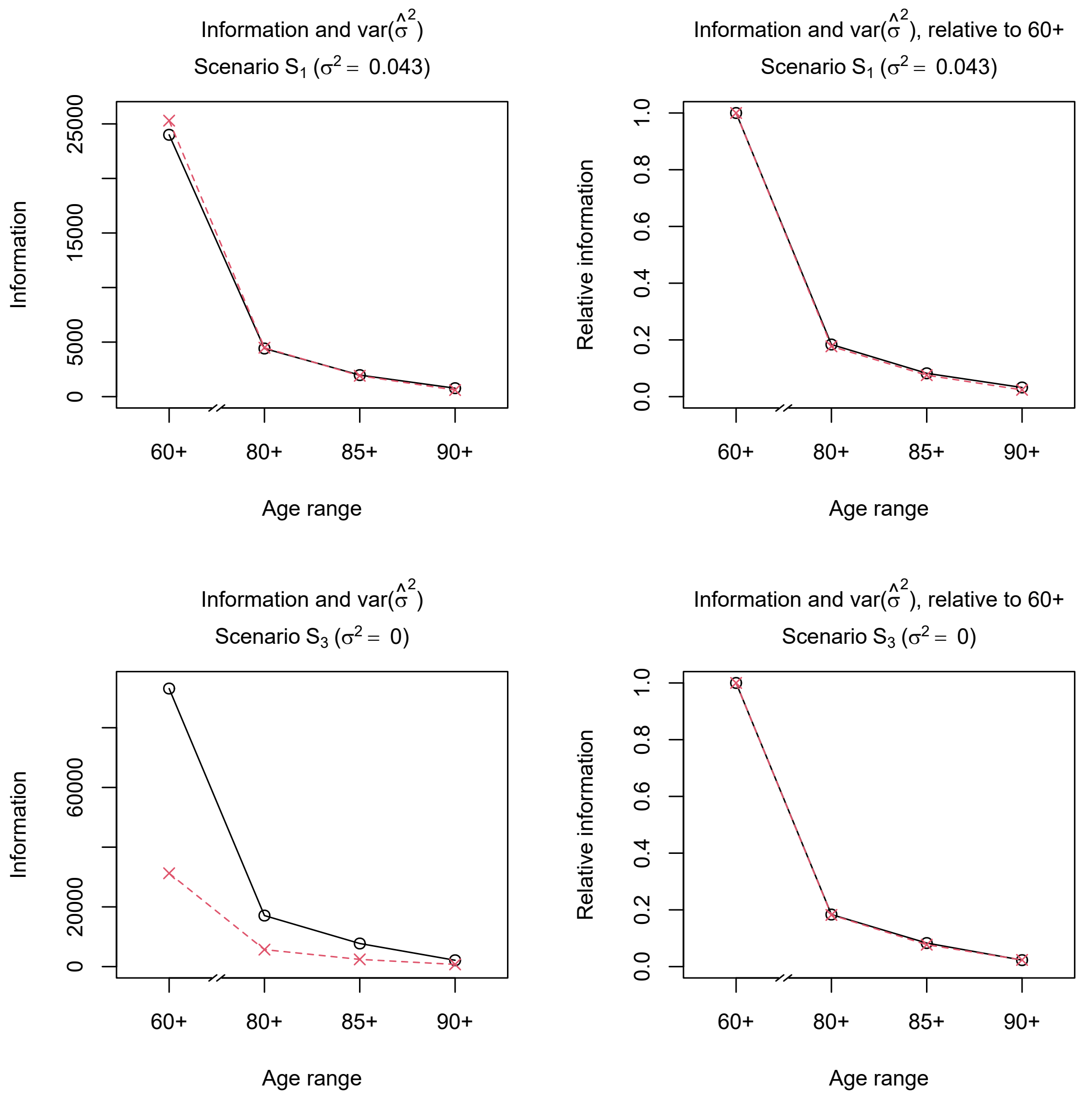

Figure S.1: Information measure $n\kappa^{-2}$ (red-dashed line, crosses) and inverse of the empirical variance of $\hat{\sigma}^2$ (black-solid line, circles) based on 1,000 samples from a gamma-Gompertz model under the medium-sized Scenarios S_1 (top) and S_3 (bottom) depending on the age range of the data (left to right: 60+, 80+, 85+, or 90+). Left: absolute values, right: relative to the value for the 60+ setting

S.3.2 Alternative information measures

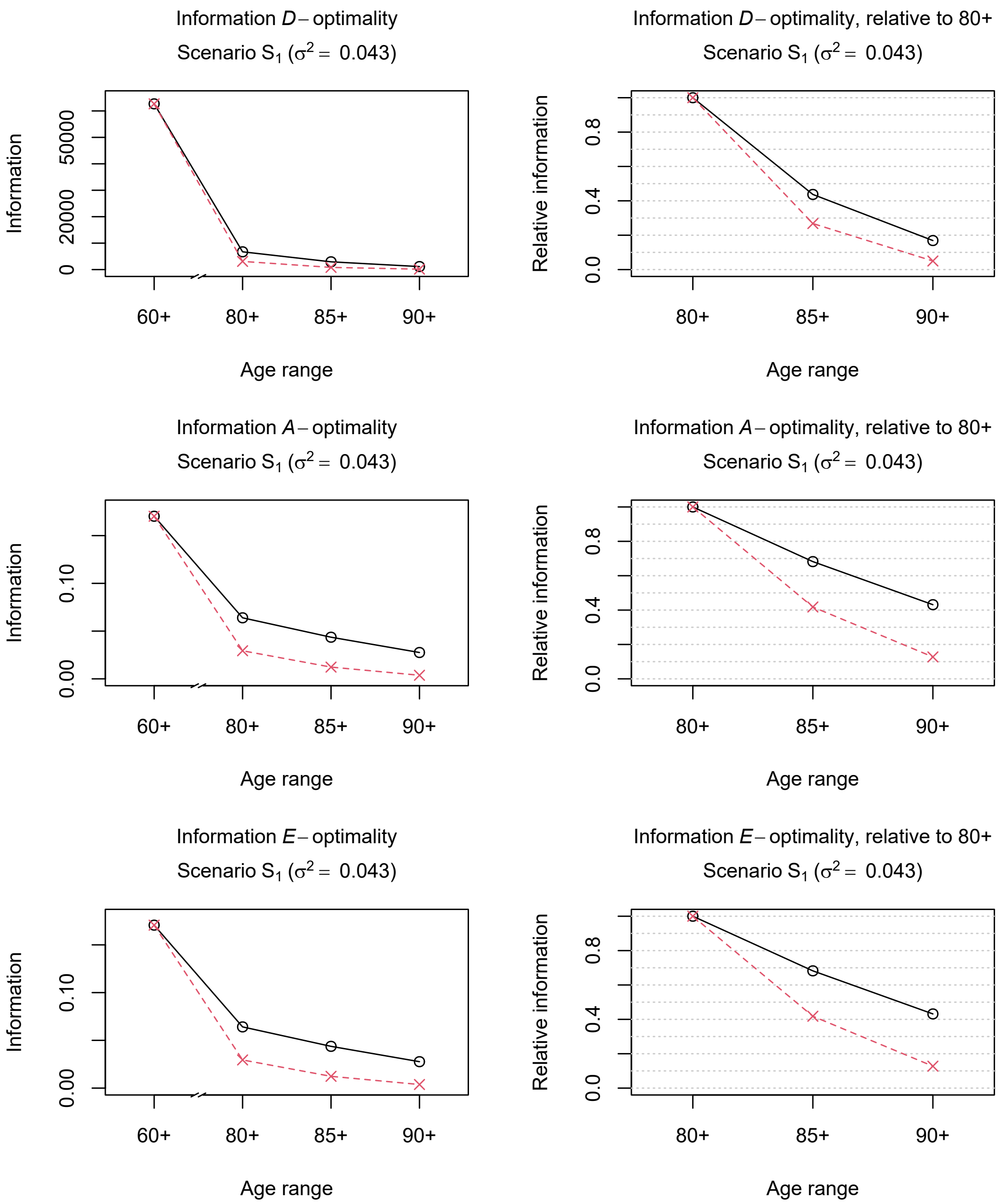

Figure S.2: Information measures $\mathcal{I}$ (black-solid line, circles) and scaled measures $\mathcal{I}^{(s)}$ (red-dashed line, crosses) under Scenario S_1 depending on the age range of the data (left to right: 60+, 80+, 85+, or 90+). Top: $\mathcal{I} = \det(\boldsymbol{I}(\boldsymbol{\theta}))$ for D-optimality, middle: $\mathcal{I} = 1/\mathrm{tr}([\boldsymbol{I}(\boldsymbol{\theta})]^{-1})$ for A-optimality, bottom: $\mathcal{I}$ as the minimum eigenvalue of $\boldsymbol{I}(\boldsymbol{\theta})$ for E-optimality. Left: absolute values of (scaled) $\mathcal{I}$, right: (scaled) ratios $\mathcal{I}_{x+}/\mathcal{I}_{80+}$ for $x = 80, 85, 90$

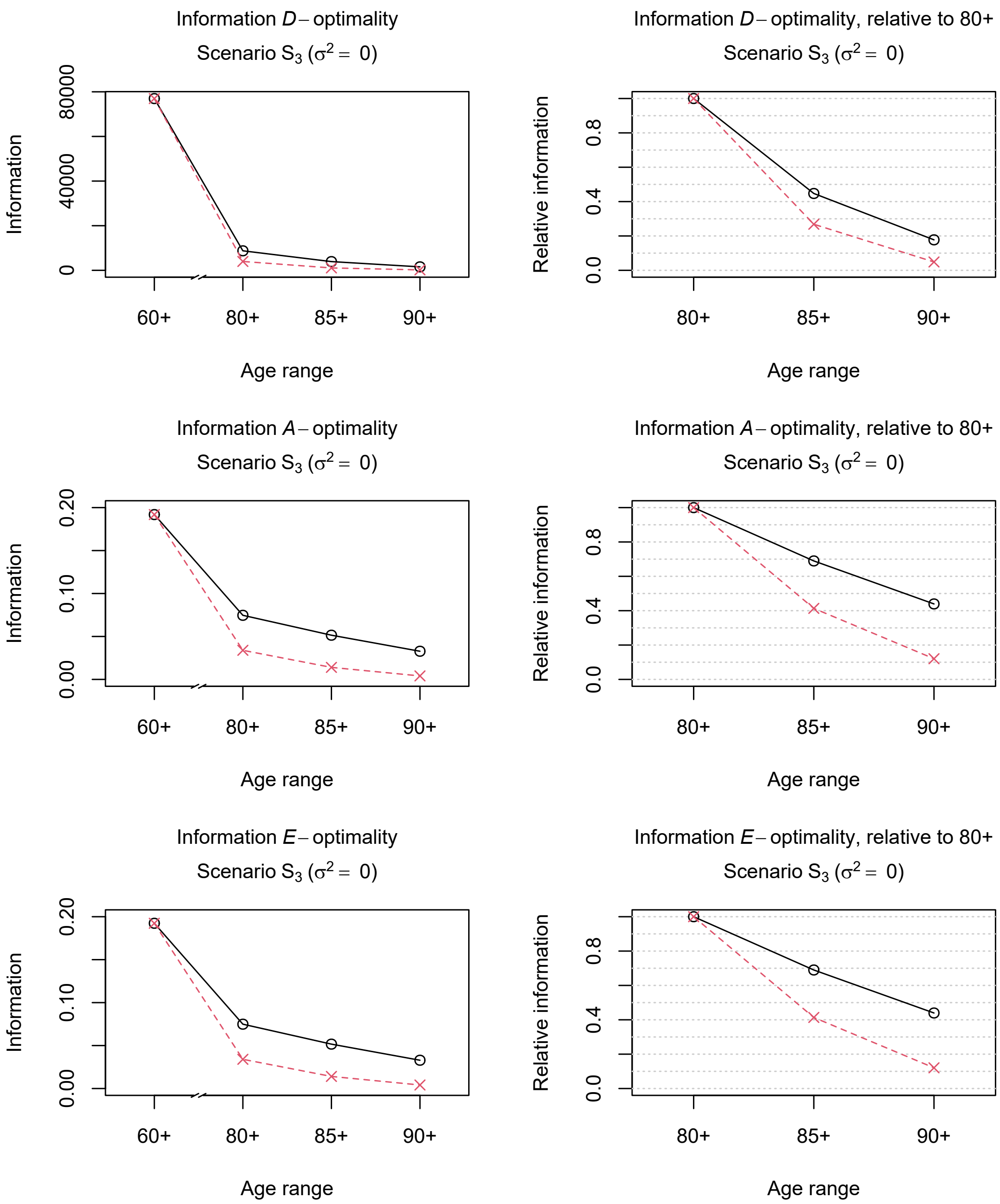

Figure S.3: Information measures $\mathcal{I}$ (black-solid line, circles) and scaled measures $\mathcal{I}^{(s)}$ (red-dashed line, crosses) under Scenario S_3 depending on the age range of the data (left to right: 60+, 80+, 85+, or 90+). Top: $\mathcal{I} = \det(\boldsymbol{I}(\boldsymbol{\theta}))$ for D-optimality, middle: $\mathcal{I} = 1/\mathrm{tr}([\boldsymbol{I}(\boldsymbol{\theta})]^{-1})$ for A-optimality, bottom: $\mathcal{I}$ as the minimum eigenvalue of $\boldsymbol{I}(\boldsymbol{\theta})$ for E-optimality. Left: absolute values of (scaled) $\mathcal{I}$, right: (scaled) ratios $\mathcal{I}_{x+}/\mathcal{I}_{80+}$ for $x = 80, 85, 90$

S.3.3 Information measures and power of the likelihood ratio test for scenarios with different values of the Gompertz parameters

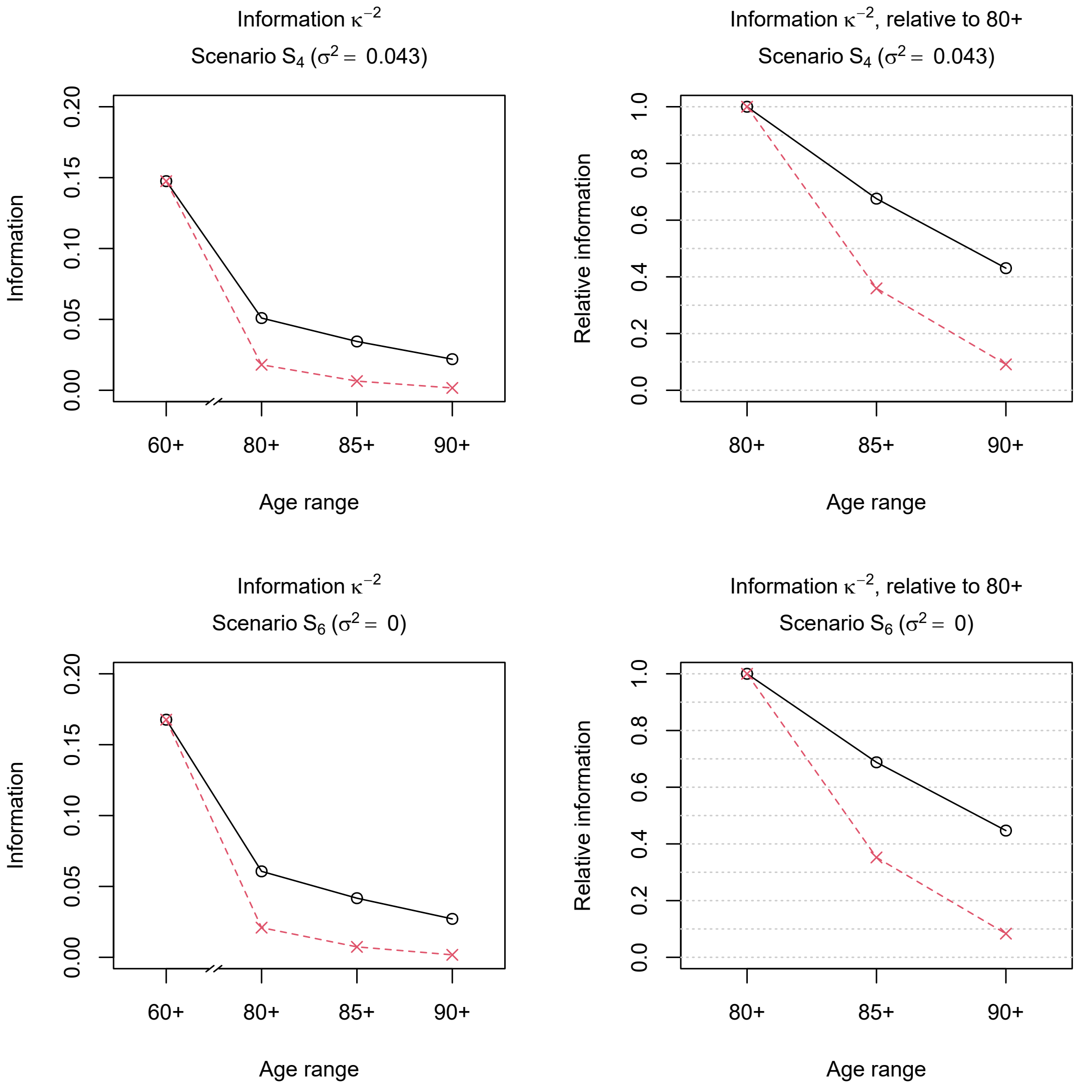

Figure S.4: Information measure $\mathcal{I} = \kappa^{-2}$ (black-solid line, circles) and scaled measure $\mathcal{I}^{(s)}$ (red-dashed line, crosses) under Scenarios S_4 (top) and S_6 (bottom) depending on the age range of the data (left to right: 60+, 80+, 85+, or 90+). Left: absolute values of (scaled) $\mathcal{I}$, right: (scaled) ratios $\mathcal{I}_{x+}/\mathcal{I}_{80+}$ for $x = 80, 85, 90$

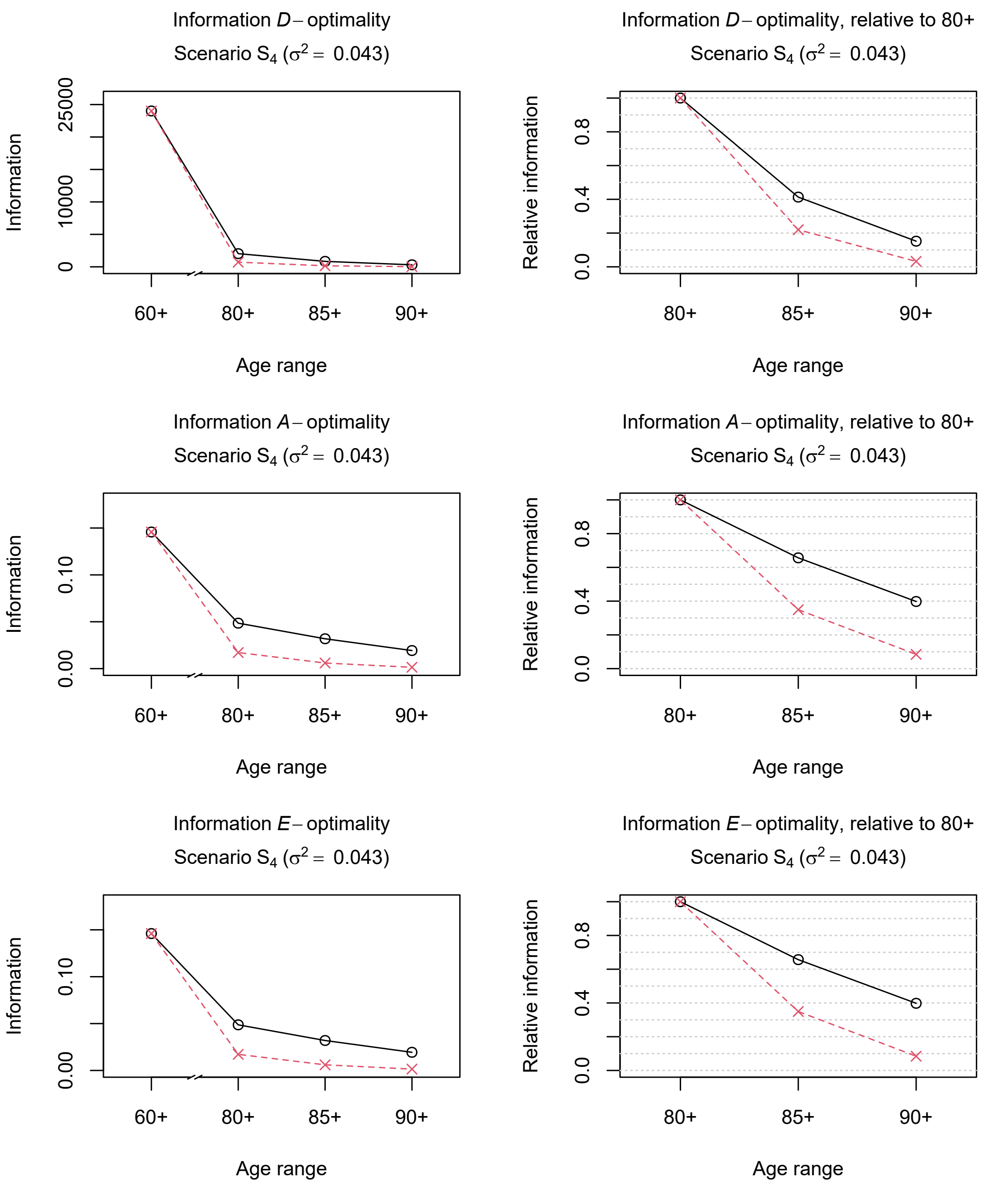

Figure S.5: Information measures $\mathcal{I}$ (black-solid line, circles) and scaled measures $\mathcal{I}^{(s)}$ (red-dashed line, crosses) under Scenario S_4 depending on the age range of the data (left to right: 60+, 80+, 85+, or 90+). Top: $\mathcal{I} = \det(\boldsymbol{I}(\boldsymbol{\theta}))$ for D-optimality, middle: $\mathcal{I} = 1/\mathrm{tr}([\boldsymbol{I}(\boldsymbol{\theta})]^{-1})$ for A-optimality, bottom: $\mathcal{I}$ as the minimum eigenvalue of $\boldsymbol{I}(\boldsymbol{\theta})$ for E-optimality. Left: absolute values of (scaled) $\mathcal{I}$, right: (scaled) ratios $\mathcal{I}_{x+}/\mathcal{I}_{80+}$ for $x = 80, 85, 90$

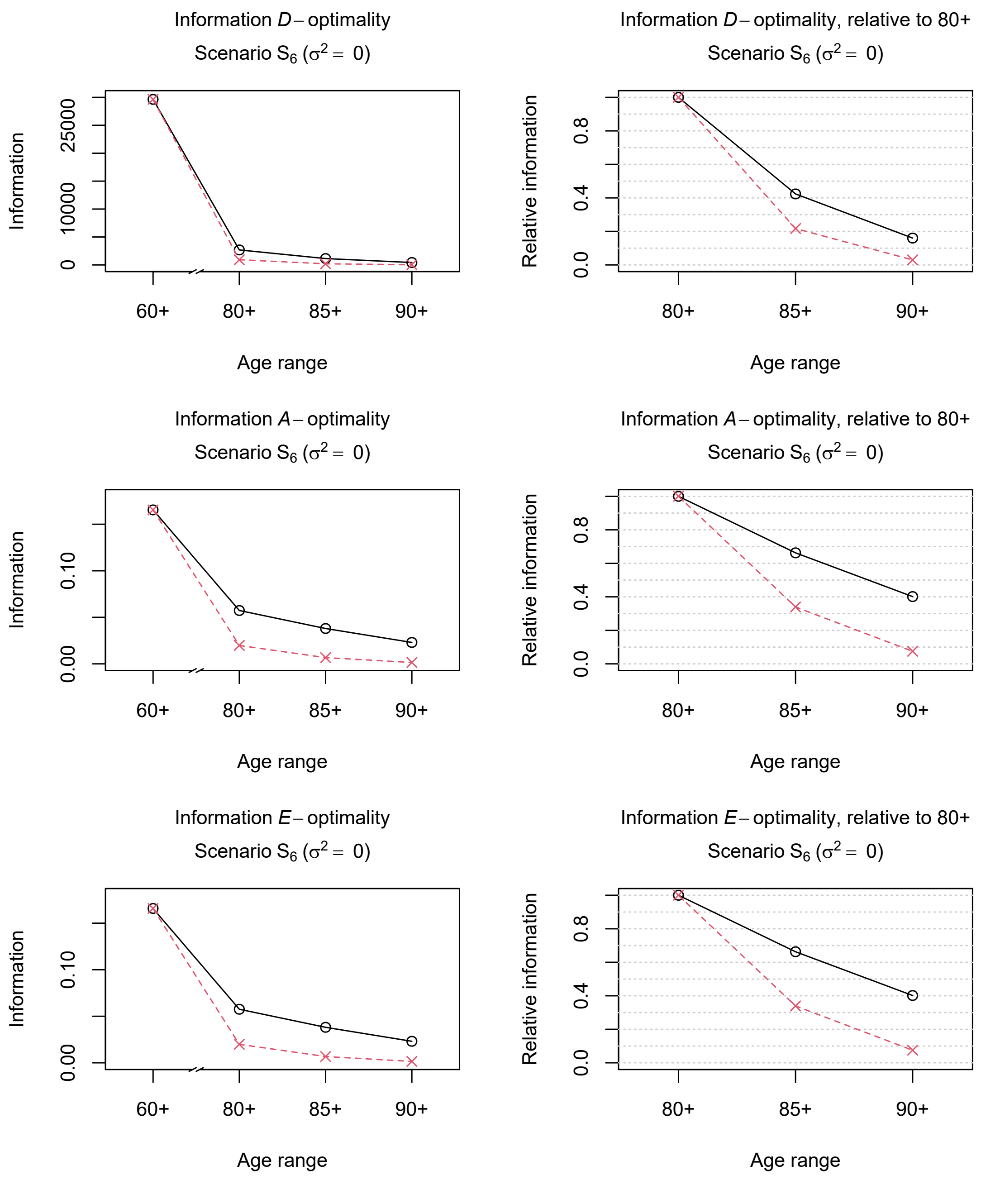

Figure S.6: Information measures $\mathcal{I}$ (black-solid line, circles) and scaled measures $\mathcal{I}^{(s)}$ (red-dashed line, crosses) under Scenario S_6 depending on the age range of the data (left to right: 60+, 80+, 85+, or 90+). Top: $\mathcal{I} = \det(\boldsymbol{I}(\boldsymbol{\theta}))$ for D-optimality, middle: $\mathcal{I} = 1/\mathrm{tr}([\boldsymbol{I}(\boldsymbol{\theta})]^{-1})$ for A-optimality, bottom: $\mathcal{I}$ as the minimum eigenvalue of $\boldsymbol{I}(\boldsymbol{\theta})$ for E-optimality. Left: absolute values of (scaled) $\mathcal{I}$, right: (scaled) ratios $\mathcal{I}_{x+}/\mathcal{I}_{80+}$ for $x = 80, 85, 90$

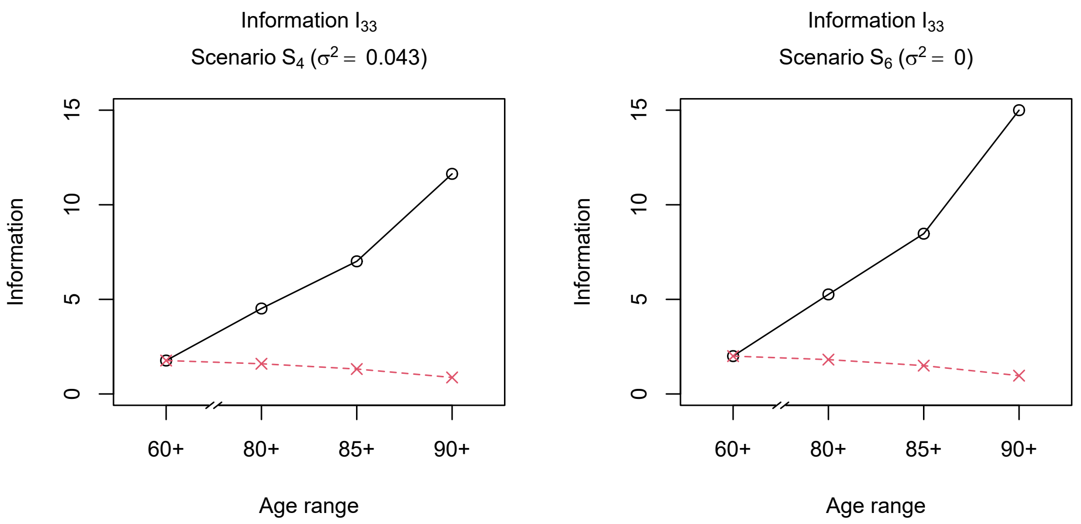

Figure S.7: Information measure $\mathcal{I} = [\boldsymbol{I}(\boldsymbol{\theta})]_{33}$ (black-solid line, circles) and scaled measure $\mathcal{I}^{(s)}$ (red-dashed line, crosses) depending on the age range of the data (left to right: 60+, 80+, 85+, or 90+) under Scenarios S_4 (left) and S_6 (right)

Table S.2: Power β of the likelihood ratio test, performed at the 5% level, according to formula (6), under Scenarios S_4 ($\sigma^2 = 0.043$) and S_5 ($\sigma^2 = 0.021$) for three sample size settings (s – small, m – medium, l – large) and varying age range

		Survivors to ages							
		60+		80+		85+		90+	
Scen.	n	n_{60+}	β_{60+}	n_{80+}	β_{80+}	n_{85+}	β_{85+}	n_{90+}	β_{90+}
S_4	s	133,506	1.000	47,165	0.678	25,090	0.352	10,000	0.157
	m	267,012	1.000	94,329	0.909	50,179	0.557	20,000	0.228
	l	1,401,813	1.000	495,229	1.000	263,441	0.993	105,000	0.662
S_5	s	143,746	0.935	50,181	0.296	26,196	0.163	10,000	0.094
	m	287,493	0.998	100,362	0.470	52,392	0.239	20,000	0.119
	l	1,509,337	1.000	526,901	0.975	275,058	0.692	105,000	0.281
